# An integrated overview of acute heart failure: a narrative review of pathophysiological mechanisms and contributing factors

**DOI:** 10.1186/s43044-026-00780-1

**Published:** 2026-09-18

**Authors:** Mohammadjavad Sotoudeheian, Mohammad Moradi, Mohammad Pirhayati, Seyed-Mohamad-Sadegh Mirahmadi, Reza Azarbad, Mohammad Sedigh Dakkali, Mehdi Taghizadeh, Hamidreza Pazoki Toroudi

**Affiliations:** 1 Physiology Research Center, Iran University of Medical Sciences, Tehran, Iran; 2 School of Medicine, Iran University of Medical Sciences, Tehran, Iran; 3 Firoozgar Clinical Research Development Center (FCRDC), Firoozgar Hospital, Tehran, Iran; 4 Cellular and Molecular Biology Research Center, Health Research Institute, Babol University of Medical Sciences, Babol, Iran; 5 Shahid Madani Hospital, Tabriz University of Medical Sciences, Tabriz, Iran; 6 Department of Physiology, Faculty of Medicine, Iran University of Medical Sciences, Tehran, Iran

**Keywords:** Heart failure, Fluid shifts, Neurohormones, Primary hypertension

## Abstract

**Background:**

Acute heart failure (AHF) is a significant cardiovascular syndrome characterized by either new-onset heart failure (HF) or acutely decompensated heart failure (ADHF), typically presenting with systemic and pulmonary congestion. The European Society of Cardiology has reinforced the importance of early guideline-directed medical therapy (GDMT) initiation and individualized decongestive strategies. This article explores current knowledge of AHF pathophysiology. The simplified hemodynamic paradigm previously used to explain AHF has been replaced over the past few decades by a more complex network of interacting mechanisms.

**Main text:**

The clinical presentation of AHF reflects a heterogeneous combination of cardiac abnormalities and systemic pathophysiological processes rather than a single disease entity. Despite overlapping clinical presentations, AHF arises from diverse underlying conditions and pathophysiological mechanisms. Left ventricular systolic or diastolic dysfunction elevates filling pressures, contributing to increased preload and afterload, which in turn promotes pulmonary congestion. End-organ dysfunction may result from the combined effects of venous congestion, impaired perfusion, and neurohormonal and inflammatory activation. Despite advances, limited progress in acute management may contribute to persistently poor outcomes in AHF patients.

**Conclusion:**

Initial management of AHF commonly includes intravenous diuretics for patients with congestion, while vasodilators and other therapies are selected according to congestion status, perfusion, blood pressure, hemodynamic profile, clinical phenotype, and the predominant underlying pathophysiological mechanism. This review examines pathophysiological processes contributing to AHF onset and progression, highlighting clinical implications and therapeutic targets.

## Background

Structural abnormalities or dysfunction of the heart characterize the clinical syndrome of heart failure (HF). There are many concepts to categorize this broad-spectrum syndrome. Based on left ventricular ejection fraction (LVEF), HF is categorized as heart failure with preserved ejection fraction (HFpEF), heart failure with mildly reduced ejection fraction (HFmrEF), and heart failure with reduced ejection fraction (HFrEF) [1, 2]. Elevated cardiac filling pressures are a consequence of cardiac dysfunction at rest and under stress [1]. The main symptoms and signs of HF are dyspnea and fatigue, abnormal pulmonary sounds, peripheral edema, and distended jugular veins. Long-term survival for patients with HFrEF has significantly improved compared with historical outcomes, though it remains worse than in patients without HF. However, the prevalence of HF has sharply increased due to demographic changes, particularly population aging and increased life expectancy [3, 4]. Since the 1990s, HF-related hospital admissions have tripled in developed countries, and HF now affects approximately 2% of the adult population [3].

The 2023 focused update of the ESC guidelines introduced key changes, including a Class I, Level A recommendation for sodium-glucose cotransporter 2 inhibitors (SGLT2i) across all HF phenotypes (HFrEF, HFmrEF, and HFpEF). This supports the use of SGLT2 inhibitors across the spectrum of left ventricular ejection fraction. The update also introduced a Class I, Level B recommendation for rapid initiation and prompt up-titration of guideline-directed medical therapy following HF decompensation [5, 6].

## Acute heart failure

Clinically, acute heart failure (AHF) refers to the rapid onset or deterioration of heart failure symptoms [1]. The majority of patients hospitalized for AHF are over 65 years of age [7]. Despite its prevalence among hospitalized patients, particularly older adults, AHF is less well understood in terms of pathophysiology and treatment than chronic heart failure. AHF requires careful diagnosis, risk stratification, and management [8].

Clinically, *de novo* HF is distinguished from acutely decompensated HF (ADHF). *De novo* HF occurs in individuals without a prior history of HF, whereas ADHF refers to worsening symptoms in patients with established chronic HF. Although this classification offers limited pathophysiological insight, it has important clinical utility. Therefore, the pathophysiology of *de novo* HF warrants more detailed investigation than that of ADHF. ADHF is more common than *de novo* AHF among hospitalizations because HF is chronic and progressive [9, 10]. Symptoms and signs of AHF are primarily related to extracellular fluid accumulation. Elevated biventricular filling pressures are responsible for this accumulation [11, 12]. In Europe and Asia, short-acting vasodilators are frequently used in conjunction with non-invasive ventilation as initial therapy for AHF [13]. Cardiogenic shock carries a tenfold higher mortality rate than AHF without shock and requires specific, urgent treatment. Cardiogenic shock is characterized by clinical signs of peripheral tissue hypoperfusion and occurs in only a minority of AHF patients [14, 15]. Reported 90-day readmission rate and 1-year mortality rates in patients with AHF are still higher than those with chronic HFrEF [16, 17].

Most patients hospitalized with HFrEF exhibit increased end-systolic and end-diastolic volumes and pressures. Elevated pulmonary venous pressures, particularly in late systole, result in pulmonary congestion [18]. Patients with elevated right-sided filling pressures may experience abdominal discomfort, anorexia, and bloating as a result of systemic venous and hepatic congestion. Symptoms of systemic hypoperfusion may result from reduced contractility and consequent decreased stroke volume. The relationship between end-diastolic pressure (EDP) and volume is also shifted upward in patients with HFpEF, resulting in severe pulmonary and systemic venous congestion [19].

The pathophysiology of HF phenotypes may differ despite these similarities at the molecular level. Findings from the Biology Study to Tailored Treatment in Chronic Heart Failure (BIOSTAT-CHF) identified highly relevant biomarkers in HFrEF patients. The study showed that these biomarkers are associated with cellular mechanisms of proliferation, cardiomyocyte function, and metabolism. Biomarkers evaluated included N-terminal pro-B-type natriuretic peptide (NT-proBNP), growth differentiation factor-15 (GDF-15), and interleukin (IL)-1 receptor type 1. In contrast, biomarker profiles in HFpEF patients are closely connected to inflammatory conditions and extracellular matrix remodeling, including integrin subunit beta-2 and catenin beta-1 [19]. Additionally, patients with AHF and mid-range ejection fraction exhibited a biomarker profile reflecting interactions between cardiac myocyte stretch and inflammatory markers [20].

## Pathophysiology

**Fig. 1:**
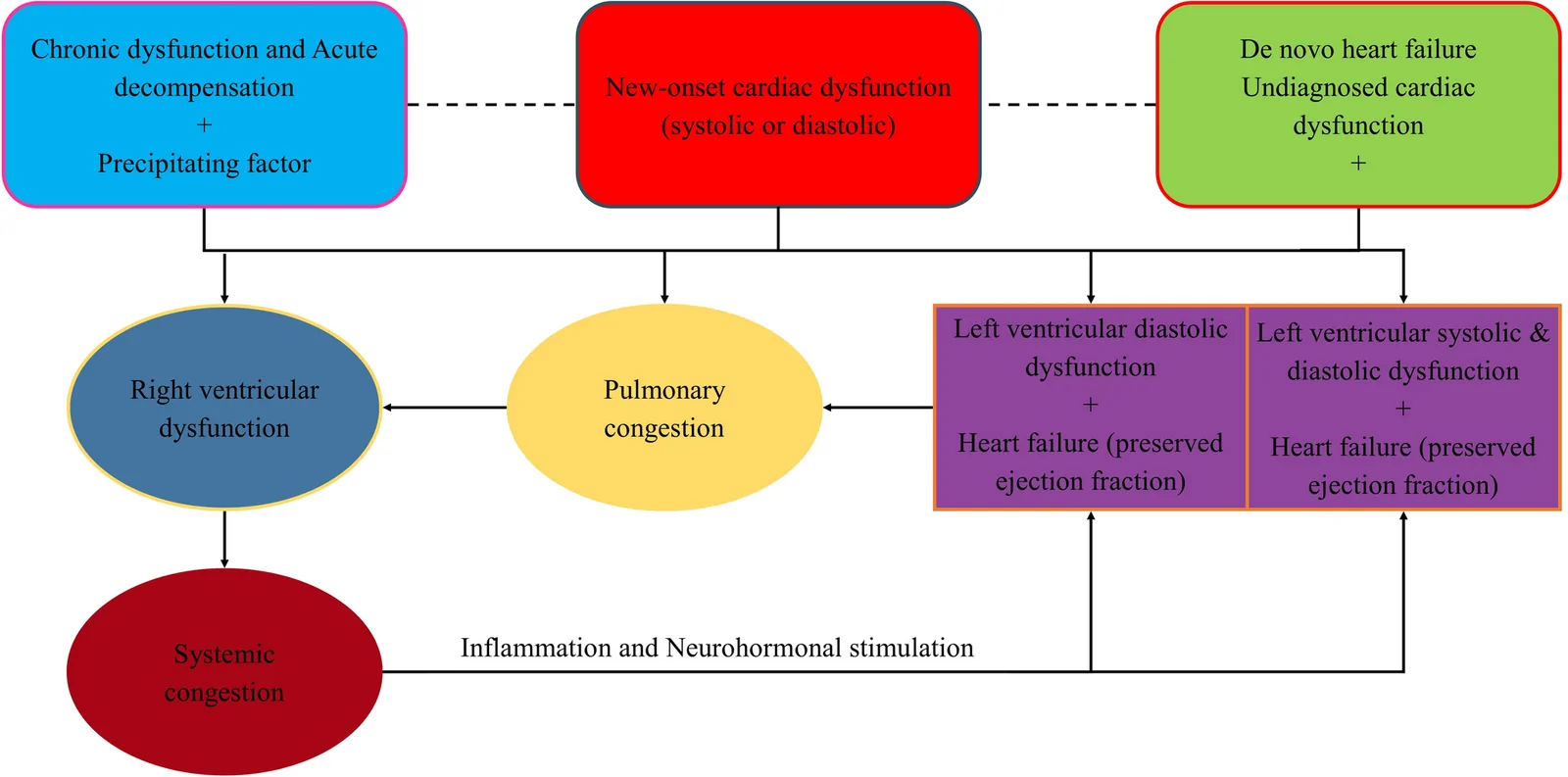
Overview of acute heart failure pathophysiology

A variety of acute and chronic cardiac pathologies can underlie AHF (Fig. 1). Acute cardiac pathologies, such as myocardial infarction, and chronic conditions, including dilated cardiomyopathy and ischemic heart disease, may both contribute to AHF. HF activates several pathophysiological pathways that counteract the deleterious effects of reduced blood flow and oxygen delivery to end organs, peripheral tissues, and cells. However, these same pathways can also contribute to systemic congestion, vascular dysfunction, ventricular remodeling, and organ dysfunction. Further, some acute diseases can precipitate AHF either through the direct impairment of cardiac diastolic function with or without impaired systolic function or by facilitating excessive accumulation of fluid in the system [21]. Most patients with AHF show systemic congestion, which is a significant contributing factor to multi-organ dysfunction. Depending on the underlying cardiac condition, AHF has a heterogeneous pathophysiology. Accordingly, different treatment strategies may be more effective in different patients depending on the underlying pathophysiology [22].

### Left ventricular systolic and diastolic dysfunction

Left ventricular (LV) diastolic dysfunction, which increases LV filling pressures and worsens pulmonary congestion, can result in AHF [23]. Acute myocardial ischemia is one example of an acute process that can produce these changes. Ischemic heart failure, LV systolic dysfunction, and pulmonary congestion are interrelated through several pathophysiological mechanisms. LV contraction requires high oxidative energy; therefore, ischemia impairs systolic performance, increasing end-diastolic volume and filling pressure. LV filling occurs in two distinct but consecutive phases: (I) an early rapid phase; in which ventricular filling depends heavily on rapid myocardial relaxation and cardiomyocyte recoil and resting stretch and (II) a later phase that depends on the late phase of left atrial contraction [24].

Atrial cardiomyocyte contraction affects the atrioventricular pressure gradient and the physical properties of the ventricle. During myocardial relaxation, cytoplasmic calcium is removed primarily by reuptake via the sarcoplasmic reticulum Ca²⁺ ATPase (SERCA) pump and partially by extrusion across the cardiomyocyte plasma membrane. In addition to structural changes or fibrosis, delayed LV relaxation affects the end-diastolic properties of the ventricle. Acute ischemia impairs myocardial relaxation with a reduction in oxidative ATP generation, which further increases filling pressures in the LV. AHF is more likely to develop in the presence of coexisting conditions such as impaired relaxation or increased end-diastolic LV stiffness [24].

Chronic LV systolic dysfunction and elevated LV end-diastolic volume, along with structural fibrosis with or without hypertrophy, may increase end-diastolic LV stiffness and increase the risk of AHF precipitated by ischemia. These abnormalities may result from systemic diseases such as diabetes mellitus, chronic hypertension, chronic kidney disease, aortic stenosis, or aging [24]. The sudden onset of atrial fibrillation or progressive impairment of atrial contractile function can also impair LV filling, significantly increasing filling pressures in patients with preexisting diastolic dysfunction. Diastolic dysfunction caused by severe mitral stenosis is more likely to cause atrial fibrillation than a structural LV disease, which could lead to AHF [22].

### Neurohormonal activation, vasoconstriction, and inflammatory crosstalk

Neurohormonal stimulation has been emphasized in the mechanistic underpinnings of HF for its fundamental role in cardiac function for decades [3, 25]. The sympathetic nervous system and renin-angiotensin-aldosterone system (RAAS) are activated early in HF, promoting compensatory mechanisms that initially help maintain perfusion. As a result, cardiac contractility may initially increase, sodium and fluid retention are increased, and peripheral vasoconstriction occurs. Chronic activation of these mechanisms leads to excessive fibroblast proliferation, oxidative stress, and extracellular matrix deposition. This may further deteriorate cardiac function and end-organ performance. Therefore, pharmacological therapies targeting the RAAS and adrenergic systems are first-line therapies for chronic HF [26, 27].

Moreover, recent studies have confirmed that neurohumoral mechanisms activated during heart failure, regardless of the ejection fraction, significantly influence cells of the immune system. These cells possess receptors for angiotensin II (AT1 receptors), adrenoceptors, and natriuretic peptides. Angiotensin II can influence macrophage polarization, promoting the M2 macrophage phenotype, which in turn, affects the T helper (Th1 and Th2) lymphocytes balance. Furthermore, the activation of monocyte β-Adrenergic Receptor (AR) is responsible for inhibiting the production of free oxygen radicals and reactive oxygen species, while the expression of tumor necrosis factor alpha (TNF-α) receptors and TNF-α release can be regulated by α2-AR. In dendritic cells, β2-AR activation inhibits interleukin (IL) 12 production, leading to the inhibition of TH1 and promoting TH2 differentiation. Atrial natriuretic peptide (ANP) activates superoxide anion secretion in polymorphonuclear cells, reduces TNF-α and nitric oxide (NO) secretion in macrophages, and attenuates exacerbated Th1 responses. In macrophages, brain natriuretic peptide (BNP) can stimulate reactive oxygen species (ROS) production, up-regulate IL-10, and inhibit IL-12 and TNF-α release by dendritic cells, suggesting an anti-inflammatory cytokine profile induction [25].

However, in the AHF setting, RAAS inhibition must be individualized based on patient condition. Neurohormonal activation is well established in chronic HF [28]. Previous studies have shown elevated levels of neurohormonal biomarkers in AHF, including plasma renin, aldosterone, norepinephrine, and endothelin-1 [29, 30].

It is believed that tissue RAAS contributes to long-term cardiac remodeling, whereas systemic RAAS can induce hemodynamic changes [31]. In addition, AHF is associated with elevated levels of inflammatory biomarkers, such as IL-6, tumor necrosis factor-α (TNF-α), and C-reactive protein (CRP), which might indicate a pro-apoptotic and prothrombotic environment [32–36]. Activated innate immune systems are also associated with increased levels of inflammatory cytokines [37].

Moreover, transmembrane receptor 2 (ST2) and galectin-3 were found to increase during inflammation, cardiac contractility failure, and cardiomyocyte stretching. They have been shown to be involved in cardiac dysfunction through modulation of fibrosis [35]. Although RAAS biomarkers are useful for evaluating disease severity, their role in predicting prognosis and their regulatory functions in AHF pathophysiology remain unclear. Nevertheless, their importance as therapeutic targets in chronic HF has been well established. However, long-term outcomes in patients with AHF following hospitalization have been associated with elevated serum aldosterone levels [38].

Loop diuretics can increase neurohormonal activity in patients with AHF [39], although some earlier studies were conducted before RAAS inhibitors were widely available. AHF is also associated with neurohormonal activation that contributes to renal function impairment (RFI) [40]. A 2014 study comparing high-dose versus low-dose loop diuretics suggested that alterations in RAAS biomarkers might be similar between groups. In each treatment group, RFI occurred in a comparable percentage of patients, regardless of the level of RAAS activation at baseline [41].

In addition, baseline levels of RAAS biomarkers may not independently predict short-term outcomes [41]. As a result of these data, we can understand how difficult it is to interpret levels of RAAS biomarkers and predict RFI when there is AHF present. The BLAST trial [42] showed that RAAS inhibition during AHF hospitalization improves surrogate markers of HF and clinical outcomes. This study provided additional evidence supporting neurohormonal modulation as a potential therapeutic strategy in AHF.

Guideline updates have reinforced the importance of neurohormonal modulation in HF management. The ESC focused update emphasizes SGLT2 inhibitors across HF phenotypes, with benefits extending beyond glycemic control to include reductions in HF hospitalization and cardiovascular events [5, 6]. The BLAST trial provided supplementary evidence supporting RAAS inhibition during AHF hospitalization, though optimal timing and patient selection remain areas of active investigation [11].

Vasoconstriction causes increased afterload, leading to hypertensive AHF. It is believed that a few specific mechanisms are especially important in acute vasoconstriction. Several stimuli have been hypothesized to contribute to acute sympathetic surge, including exercise-induced muscle metaboreflex activation [43], arterial baroreceptor insensitivity, sympathomimetic drug abuse, abrupt rebound from sympatholytic medication noncompliance, and psychosocial stress or anxiety. The activation of beta-adrenergic receptors in the heart increases contractility, while alpha-adrenergic receptors in the vascular system increase peripheral vascular resistance. In addition, angiotensin II and vasopressin may modulate the central sympathetic outflow as well, causing additional vasoconstrictor effects via the renin-angiotensin-aldosterone system [44].

Direct endothelial dysfunction may acutely reduce vascular compliance through increased oxidative stress. A proinflammatory state, triggered by infection or other stressors, causes cytokine release and inflammatory biomarker surges that sequester nitric oxide (NO), impairing vasodilation. Enhanced mobilization of blood volume toward the central circulation may result from these systemic effects, which are both venous and arterial in nature [45]. It is well understood that pulmonary artery pressure and thoracic bioimpedance increase before an episode of AHF, which is consistent with current understandings of hemodynamic changes [46].

Inflammatory biomarkers, such as CRP, have been linked to decreased vascular compliance, and higher arterial stiffness (pulse wave velocity (PWV) and aortic and brachial pulse pressure) [47]. This results in reduced cardiac reserve, exercise intolerance, fluid volume sensitivity, and hypersensitivity to sympathetic activation due to increased arterial elastance (Ea) and end-systolic elastance (Ees) [48].

### Congestion mechanisms

#### Fluid dynamics: accumulation, redistribution, and intravascular congestion

Intravascular congestion is directly associated with the signs and symptoms of ADHF [49]. Dietary indiscretion or renal dysfunction can contribute to the progressive accumulation of fluid. Rapid redistribution of intravascular fluid volume from peripheral vascular beds and splanchnic venous circulation may also result in amplified left ventricular filling pressures. This may increase central and pulmonary venous congestion [22, 50]. There is a multitude of positive feedback interactions that worsen ADHF as the intravascular fluid volume expands or redistributes [51].

Neurohormonal activation can promote vascular redistribution and fluid translocation, resulting in central blood volume accumulation in the central venous system, resulting in elevated central venous pressure (CVP). This increased venous congestion can decrease renal function. It is also believed that this can cause an increase in sodium and fluid retention, which further contributes to the increase in intravascular volume and ventricular preload. In patients with HFpEF and HFrEF, diastolic dysfunction is common; increased preload raises end-diastolic pressure (EDP), leading to higher ventricular wall stress and myocardial oxygen demand, which further impairs diastolic function [52].

Increased ventricular volume may exacerbate functional mitral or tricuspid regurgitation, which can further aggravate congestion and impair organ function. This cascade provides multiple potential intervention points. In addition to congestion, valvular disease, particularly mitral regurgitation (MR), should also be considered when evaluating congestion causes. MR increases pulmonary pressures and causes retrograde blood flow [52].

Primary MR may result from structural abnormalities, while secondary MR may arise from left ventricular dysfunction, right ventricular or left atrial dilation, or mitral apparatus effects [53]. MR in patients with HFrEF is associated with poor clinical outcomes [54–56].

To treat the clinical manifestations of intravascular fluid accumulation and congestion, diuretics and vasodilators may be used according to the patient’s hemodynamic and clinical profile. Treatment selection can be challenging because the predominant mechanism is often difficult to identify. Patients admitted with ADHF may benefit from invasive assessment with right heart catheterization to guide aggressive diuresis and vasodilator treatment [27]. In patients with moderate to severe MR, reevaluation of MR should be performed once euvolemia is achieved to determine whether transcatheter or surgical intervention is indicated. Despite proven clinical improvement, hemodynamic congestion often persists even when clinical congestion has subsided. AHF patients who are inadequately treated with diuretics before discharge are more likely to develop recurrent congestion and require frequent hospitalizations. Hemodynamic congestion may precede overt clinical congestion by several weeks and may remain clinically silent. An association between raised filling pressures and cardiovascular events has been confirmed by continuous pulmonary artery pressure monitoring [57].

Extracellular fluid volume and venous bed compliance can contribute to elevated filling pressures in HF. AHF frequently occurs in patients with underlying diastolic dysfunction, which is accompanied by fluid accumulation with or without redistribution, resulting in systemic congestion [58]. Changes in central intravascular volume are not linearly related to changes in interstitial fluid volume [59]. Studies have shown that low sympathetic activity does not increase cardiac filling pressures even when intravascular volume expands significantly [60, 61].

Diuretic therapy may only marginally reduce intravascular volume in HF patients despite significant weight loss [59]. However, changes in venous bed compliance are also a major determinant of congestion in approximately half of patients presenting with ADHF within the month before admission [62]. Both the intravascular and interstitial compartments store most of the retained sodium [63]. As interstitial glycosaminoglycan networks buffer sodium without retaining water, increased interstitial sodium storage does not necessarily cause edema in healthy individuals [64].

Glycosaminoglycan networks in the interstitial space show low compliance as well. As a result, fluid accumulation is prevented in the interstitium [65]. Even mildly elevated hydrostatic pressure can cause edema formation in subjects with any manifestations of HF while the extracellular glycosaminoglycan networks are disrupted due to sodium accumulation [63]. Fluid accumulation is often associated with increased neurohumoral activity leading to renal water and salt retention, but it may also be iatrogenic. Patients with chronic HF or renal disorder are particularly prone to fluid accumulation due to the activation of the neurohumoral pathway early in the progression of their disease. Neurohumoral activation mechanisms and consequences have been thoroughly reviewed elsewhere [66]. This self-perpetuating process is exacerbated by organ dysfunction. As a result of alterations in both the distal and proximal segments of the nephron in HF, the renal sodium avidity increases even before clinical symptoms appear [67–69].

Decreased natriuresis in RFI has also been linked to elevated CVP in several studies [70–72]. Assessment of changes in renal function during AHF helps stratify risk and guide treatment strategies. AHF may cause apparent RFI even with favorable diuresis and improved HF status, as renal function parameters may temporarily worsen. Decongestion in AHF has been hindered by the misinterpretation of RFI. Renal function assessment in AHF must evaluate not only measures of glomerular filtration, including estimated glomerular filtration rate (eGFR) but also tubular response to diuretics, including the ability to eliminate residual fluid, to distinguish true RFI from pseudo-RFI [22].

In response to sympathetic stimulation, the peripheral venous system and the splanchnic system can suddenly shift volume to the pulmonary circulation without the involvement of exogenous fluids. This results in fluid redistribution [73]. Large veins physiologically contain approximately one-quarter of total blood volume, helping regulate fluid retention and stabilize cardiac preload [74].

End-diastolic volume and pressure are correlated with the compliance of myocardium during preload. In contrast, the afterload represents the pressure against which the ventricle must contract to eject blood. Afterload is closely related to systolic arterial pressure. Therefore, increased afterload combined with reduced venous capacitance may cause ventricular-vascular uncoupling, resulting in systemic, organ, and/or pulmonary congestion [75]. Additionally, acute mechanical factors might similarly cause AHF by increasing ventricular preload. It is also possible to develop a ventricular septal defect or rupture of the papillary muscles or chordae tendineae suddenly, leading to mitral valve regurgitation. Decongestive therapy should vary according to the clinical scenario, as fluid accumulation and fluid redistribution contribute to systemic organ congestion in AHF [21].

Global End-Diastolic Volume (GEDV), Right Ventricular End-Diastolic Volume (RVEDV), and Stroke Volume Variation (SVV) may provide useful estimates of intravascular volume status of intravascular volume status. These volumetric parameters are unaffected by the intrathoracic pressure elevations induced by intra-abdominal hypertension (IAH), which otherwise cause erroneously high Pulmonary Artery Occlusive Pressure (PAOP) and Central Venous Pressure (CVP). While calculating transmural pressures (e.g., Transmural PAOP = PAOP − 0.5 × IAP) can offer some correction, these volumetric measures provide a more accurate assessment of volume status, even when IAP is as low as 10 mmHg [76].

Elevated IAP is a significant, often overlooked, factor contributing to increased morbidity and mortality, particularly among critically ill patients. In conditions like AHF, where systemic venous congestion is common, sustained IAH can significantly impair organ perfusion and cardiac function [77]. Rising IAP directly increases intrathoracic pressure, thereby reducing venous return to the heart and decreasing cardiac output. This effect is particularly pronounced even at mild IAP elevations (as low as 10 mmHg) and can be exacerbated in hypovolemic states. Furthermore, IAP contributes to increased systemic and pulmonary vascular resistance, acting as a significant afterload that can be poorly tolerated by compromised hearts. Paradoxically, intracardiac filling pressures like PAOP and CVP may appear elevated due to increased intrathoracic pressure, masking true intravascular volume status. Understanding these complex pathophysiologic mechanisms of IAP is fundamental for recognizing IAH, effectively managing patients, and preventing end-organ dysfunction, which is crucial in the critically ill context of AHF [76].

Systemic congestion may develop and progress before overt AHF becomes clinically apparent. Several factors can trigger it, either directly or indirectly, over the course of hours or days. These mechanisms cause fluid accumulation or redistribution, either directly or indirectly through worsening diastolic and/or systolic function. Understanding the underlying pathophysiology is essential for appropriate AHF treatment. AHF may occur without a substantial increase in body weight in a considerable proportion of patients, despite a growth in pulmonary pressures prior to hospitalization. AHF patients with precipitating factors have been examined by a variety of registries, such as the North American OPTIMIZE-HF registry and the Euro-Asian GREAT network registry [78, 79].

The most common precipitants include acute coronary syndromes, arrhythmias, infections, poorly controlled hypertension, and nonadherence to dietary recommendations or medications. About 5–20% of patients showed a combination of multiple precipitants, whereas 40–50% had no precipitants identified [78, 79].

Precipitating factors are associated with higher mortality and readmission rates [78–81]. Short-term mortality rates are higher for AHF precipitated by acute coronary syndromes or infection than for AHF precipitated by atrial fibrillation or uncontrolled hypertension [78, 79].

Although prognosis is similarly unfavorable in AHF precipitated by acute coronary syndromes or infection, the mortality risk changes differently over time: it peaks immediately after admission in the first group, but approximately three weeks after admission in the latter [79, 82]. This phenomenon may reflect complex interactions between infection, endothelial dysfunction, inflammatory and ischemic myocardial injury, plaque instability, coagulation activation, fluid retention, arrhythmias, and other precipitating factors [79]. Additionally, by identifying precipitating factors, specific treatments can be implemented to treat AHF’s underlying causes and not just decongestive therapy [22].

#### Venous congestion and endothelial dysfunction

Activation of neurohormones, aggravation of renal failure, and endothelial cell activation contribute to venous congestion’s underlying pathophysiology in AHF. Patients may experience AHF symptoms differently as a result of venous congestion. Some patients with prominent right-sided cardiac dysfunction develop ascites and peripheral edema due to retrograde congestion into hepatic vessels and tissues [83]. These patients commonly experience progressive volume overload before AHF hospitalization [83].

Alternatively, many patients with AHF do not gain substantial weight or develop symptoms rapidly before presenting. It has been speculated that these patients develop symptoms of vascular congestion due to rapid fluid translocation from sympathetic activity-mediated splanchnic vasoconstriction [61]. When hypoxia occurs intermittently, the sympathetic nervous system is activated, resulting in a shift of blood into the effective circulating volume [84].

Fluid overload causes neurohormonal activation through a positive feedback cycle, regardless of the cause of venous congestion. Apoptosis and fibrosis may occur downstream as a result of angiotensinogen expression. Venous congestion causes subendocardial ischemia and adverse ventricular remodeling through sustained myocardial stretch and local RAAS activation [44]. AHF is consequently associated with cardiac dysfunction and declining renal function [70, 85]. Apoptosis occurs through the interplay between Bcl-2 family proteins [86, 87].

As congestion and renal function deteriorate, loop diuretics’ dose-response curves shift rightward and downward, and higher therapeutic doses are required to preserve sufficient diuresis. Renal adaptations and escape mechanisms may also contribute to diuretic resistance. Examples include increased prorenin activity and aldosterone breakthrough. However, these mechanisms do not appear to be associated with positive outcomes [88]. In cardiorenal syndrome types 3 and 4, renal dysfunction may be acute or chronic. Additionally, electrolyte imbalance, acid-base disturbances, sympathetic activation, chronic or acute hypertension, inflammation, and myocardial ischemia (with or without reperfusion) are associated with worse cardiac function [89, 90]. Multiple factors contribute to the physiological alterations seen in ischemia-reperfusion injury [91].

The function of venous endothelial cells is also impaired by congestion-mediated vascular stretch [92]. There is also evidence that endothelial dysfunction contributes to HF development and progression [93]. Endothelial NO release and metabolism, along with hormone-like substances, prostaglandins, and inflammatory cytokines, affect myocardial function, hemodynamics, coronary blood supply, and renovascular circulation [94]. Acute endothelitis in AHF is characterized by severe impairment in endothelium-dependent NO vasodilation [45].

Cardiorenal syndrome is also associated with impaired NO bioavailability and increased oxidative stress [40]. Oxidative stress plays a pivotal role in heart failure caused by ischemia, making certain pathways promising targets for potential treatment [95, 96]. Researchers have shown that elevated venous arm pressure in healthy individuals alters the expression profiles of endothelial cells and activates neurohormones [97]. As a result of these findings, the pathophysiology of AHF appears to include both venous congestion and endothelial dysfunction.

Endothelial dysfunction, which is caused by oxidative stress, is a contributing factor in the development of heart failure. The pathophysiology of both HFrEF and HFpEF involves changes in endothelial function and the nitric oxide-cyclic guanosine monophosphate (NO-cGMP) pathway. When endothelium-dependent vasodilation is altered, it can result in repeated episodes of ischemia/reperfusion, leading to a chronically stunned myocardium with systolic dysfunction and increased diastolic stiffness, causing diastolic dysfunction. Additionally, an altered NO-cGMP pathway can directly affect myocardial homeostasis. Endothelial dysfunction is associated with a worse prognosis and a higher rate of cardiovascular events. The NO-cGMP pathway therefore represents a potential therapeutic target in HF. Although clinical data remain inconclusive, this pathway warrants further investigation [98, 99].

#### Congestion and organ dysfunction

When ventricular filling pressures are elevated, ventricular wall tension increases, and myocardial stretching and remodeling occur, which contribute to impaired cardiomyocyte contractile function or overall cardiac contractility, valvular insufficiency, and end-organ or systemic congestion [100]. There is a compensatory release of circulating natriuretic peptides by the atrial and ventricular cardiomyocytes to counteract the increased wall tension, and a high proportion of patients with AHF have detectable or elevated high-sensitivity cardiac troponin levels, reflecting myocardial injury that may occur even in the absence of overt ischemia or necrosis [101].

Elevated left atrial pressure and mitral regurgitation increase pulmonary capillary hydrostatic pressure, causing lung stiffness and dyspnea [102]. There are other mechanisms involved in fluid homeostasis in addition to hydrostatic pressure. The lymphangiogenic factor vascular endothelial growth factor-D (VEGF-D) has been associated with fluid homeostasis in HF and renal failure [103–105]. While the lymphatic system is capable of draining large volumes of interstitial fluid in the early stages of lung congestion, eventually the capacity is exceeded. This leads to pleural effusions and pulmonary edema when fluid moves into the pleural and alveolar spaces [106].

Systemic congestion is present in most patients with AHF [11]. A number of organs contribute to congestion development and propagation, along with poor cardiac function [107]. Hypoperfusion might contribute to further deterioration of organ function and is associated with increased mortality risk in AHF. Congestion is an important contributor to organ dysfunction in AHF [11]. In patients with AHF, improving organ function through decongestive therapies reduces death risks. Therefore, preventing and treating organ dysfunction is a key therapeutic objective [22] .

HF patients with chronic or acute venous congestion experience significant organ dysfunction. It is known as cardiorenal syndrome when cardiac dysfunction occurs closely with renal dysfunction [89]. As a result of decreased cardiac index and arterial underfilling in HF, renal dysfunction is characterized by hypoperfusion [108]. In AHF, the low cardiac index alone has only minor effects on renal function, while venous congestion was the most vital hemodynamic determinant of renal dysfunction. Elevated CVP indicates venous congestion [70, 85].

Low cardiac index and elevated CVP are particularly detrimental to renal function. Visceral congestion in HF may increase intra-abdominal pressure, further impairing renal function. Recent research shows that decongestive therapy can alleviate renal and abdominal congestion, decreasing serum creatinine levels. Cardiac dysfunction negatively influences prognosis in AHF patients [109, 110]. Elevated transaminase levels in AHF indicate hypoxic hepatitis due to hypoperfusion, while cholestatic liver dysfunction occurs most commonly in HF [111, 112]. Finally, advanced HF patients may develop cachexia from intestinal congestion [113] .

#### Renal dysfunction in AHF

Cardiorenal syndrome is an example of heart-kidney interdependence [89, 90]. Patients with chronic HF are at increased risk for adverse events due to renal dysfunction [114, 115]. Renal dysfunction adversely impacts in-hospital and post-discharge outcomes in AHF patients [116]. It has also been found that RFI during hospitalization for AHF portends a poor prognosis [117–120]. However, transient deterioration of renal function may not necessarily lead to adverse outcomes [121, 122]. Outcomes may remain favorable when adequate decongestion is achieved despite transient worsening of renal function [122].

In AHF, renal dysfunction is driven by several interrelated pathways [40, 89]. Three primary mechanisms underlie baseline renal dysfunction and the development of RFI during AHF hospitalization: venous congestion, neurohormonal activation, and inflammation. However, the specific mechanisms underlying renal dysfunction remain poorly understood [123].

Several studies have suggested that venous congestion may contribute more strongly to renal dysfunction than reduced cardiac output in AHF [70, 85]. However, subsequent data have challenged venous congestion as a unifying or isolated mechanism [123]. RFI was found not to be associated with baseline and change in venous pressures in one study assessing renal function and hemodynamic measures [124]. In addition to volume, venous tone and sympathetic activation may influence venous congestion [61, 123]. As a result, while current studies have focused largely on venous congestion, renal dysfunction may have neurohormonal, inflammatory, or both underlying mechanisms [11].

Elevated CVP increases renal venous pressure, raising interstitial tissue pressure in the kidneys. This causes renal interstitial hydrostatic pressure to exceed intratubular pressure, leading to tubule collapse and decreased GFR [51]. Renal venous hypertension also causes renal hypoxia, ultimately leading to interstitial fibrosis [70, 85, 125]. An increase in intra-abdominal pressure, impaired cardiac output, and inflammation may all contribute to renal dysfunction caused by AHF [12, 126].

Therefore, excessive or poorly targeted decongestion may contribute to tubular stress or injury. Consequently, clinicians often interpret rising plasma creatinine as hypovolemia, leading to reduced decongestive therapy. However, as discussed above [127, 128], this is not always the case. Decongestive therapy should be continued until euvolemia is achieved in patients with rising creatinine [129], because persistent congestion at discharge is associated with poor clinical outcomes [130]. Conversely, patients without significant residual congestion may be overtreated with loop diuretics if only serial natriuretic peptide levels are used to assess volume status. Dose escalation may cause adverse effects including RFI. In patients with HF, evaluating congestion multidimensionally before discharge would be beneficial. In addition to biomarkers, clinical assessments at rest and during dynamic maneuvers should be combined with echocardiography and pulmonary pressure measurements, though prospective studies are needed [129].

Liver congestion may be associated with elevated alkaline phosphatase, bilirubin, and gamma-glutamyl transferase [130–133]. In severe hypoperfusion states such as cardiogenic shock, centrilobular necrosis and markedly elevated transaminases occur [132]. Increased intra-abdominal pressure and villous ischemia from splanchnic congestion may contribute to chronic inflammation and malnutrition by altering intestinal morphology, nutrient absorption, and the gut microbiome [57, 134, 135]. Venous congestion and hypoperfusion impair splanchnic microcirculation, increasing the risk of bowel ischemia. This promotes a proinflammatory environment by allowing gram-negative bacterial lipopolysaccharide (endotoxin) to enter the circulation [75]. Congestion itself also promotes inflammatory conditions by activating endothelial cells [97, 136].

Microcirculation plays an important role in AHF. The imbalance between lactate production and clearance reflects a fundamental disturbance in microcirculatory function. Elevated lactate levels can occur even without overt peripheral hypoperfusion, suggesting subclinical microcirculatory abnormalities and tissue oxygen deficit. Several mechanisms may contribute to lactate accumulation, including low cardiac output, elevated CVP, peripheral vasoconstriction, sympathetic activation, and hepatic or renal impairment. These factors reduce oxygen delivery and limit lactate clearance. In a study of 237 AHF patients, elevated lactate (≥ 2 mmol/L) on admission was observed in 43% of cases, despite all having a warm hemodynamic profile. This indicates that microcirculatory failure may exist independently of global hypoperfusion and can promote systemic metabolic stress. Higher admission lactate was linked to subsequent renal stress, as shown by early rises in plasma neutrophil gelatinase-associated lipocalin (NGAL) even with stable creatinine. Lactate elevation may therefore serve as a sensitive marker of subclinical renal and microcirculatory dysfunction, identifying AHF patients at risk for poor outcomes [137].

The relationship between renal function changes (assessed by creatinine or eGFR) and AHF is complex. Renal function can commonly worsen or improve in AHF, affecting approximately 30–50% of heart failure patients who are admitted to the hospital. It is important to have a thorough understanding of the pathophysiological factors that contribute to changes in renal function during AHF, despite the clear link between changes in renal function and outcomes. Not all changes in eGFR are equivalent, and it is crucial to interpret them appropriately in order to enhance heart failure treatment [138].

The ESC focused update introduced a Class I, Level B recommendation for rapid initiation and up-titration of GDMT following HF decompensation [5, 6]. This approach recognizes that early optimization of foundational therapies, rather than sole reliance on diuretics, may improve renal outcomes and reduce congestion.

#### Multi-organ dysfunction

AHF presents a complex and heterogeneous pathophysiological landscape, transcending the traditional view of a purely cardiac disease. Current understanding recognizes AHF as a systemic syndrome where intricate interactions between various organ systems are associated with clinical outcomes. The underlying mechanisms are multifaceted, involving impaired hemodynamics, profound neuroendocrine and proinflammatory activation, oxidative stress, persistent myocardial ischemia, unresolved congestion, and impaired tissue perfusion. These interconnected pathophysiological processes converge to induce dysfunction or injury in peripheral organs, thereby compounding the clinical challenge. Historically, multi-organ involvement in AHF has been inadequately explored, with a tendency to investigate single organ dysfunction in isolation. However, emerging evidence from critical care medicine underscores the importance of a multi-organ perspective as a key prognosticator and guide for clinical decision-making. This perspective supports a more integrated approach to the assessment and management of AHF, recognizing the systemic nature of the disease and the critical interplay between affected organs [139].

A study by Zymlinski and colleagues [139] significantly advanced our comprehension of this systemic perspective by investigating the prevalence, clinical characteristics, and prognostic implications of an interplay between different end-organ dysfunctions in a cohort of 284 consecutive AHF patients. Their meticulously defined criteria for myocardial injury (troponin I (TnI) > 0.056 ng/mL), renal dysfunction (eGFR < 60 mL/min/1.73 m²), and liver injury/dysfunction (elevated AST/ALT, bilirubin, or low albumin) allowed for a precise stratification of patients based on the number of affected organs. Findings were striking: on admission, significant cardiac, renal, and hepatic dysfunction/injury affected 38%, 50%, and 54% of patients, respectively. Critically, patients were classified into groups with 0, 1, 2, or 3 organ injuries, with these groups comprising 17%, 36%, 35%, and 12% of the cohort, respectively. This detailed classification revealed a clear dose-response relationship between the number of affected organs and adverse outcomes [139].

The prognostic implications of multi-organ dysfunction on admission are profound. The study clearly demonstrated that multiple end-organ dysfunctions identify patients at highest risk of poor outcomes. Patients with three organ dysfunctions had a 46% 1-year survival rate (hazard ratio (HR) 6.75 compared to those without organ dysfunction). Those with two organ dysfunctions also faced significantly increased mortality (67% survival, HR 3.54), while even one organ dysfunction showed a trend towards worse outcomes (84% survival, HR 1.58). Furthermore, the study corroborated that worsening heart failure within the first 48 h was more frequent in patients with multi-organ involvement (37–38% for two or three organs) compared to those with single or no organ dysfunction (21–23%). These findings strongly advocate for a paradigm shift in AHF management, emphasizing the need to assess organ dysfunction to improve patient stratification and personalize therapy beyond traditional hemodynamic targets [139].

### Myocardial injury

In HF, cardiac troponin levels are routinely measured to assess myocardial injury [140]. Even in patients without clinical ischemia, serum troponin levels are elevated in most patients with AHF. Contributing factors include increased wall pressure, oxidative stress, inflammatory processes, neurohormonal activation, and impaired calcium handling [140]. In the setting of AHF, there have been a number of conflicting studies regarding the predictive utility of troponin. Numerous studies have demonstrated an adverse short-term outcome associated with positive or elevated troponin at baseline or after conversion from a negative to a positive level [141, 142].

However, no association was found between the baseline troponin level and post-discharge outcomes at 30 or 180 days in a study utilizing a contemporary high-sensitivity troponin test [143]. Thus, the mechanisms underlying myocardial injury in AHF remain unclear. Notably, serelaxin, a new pharmacological agent for acute heart failure, was found to reduce mortality and troponin levels at 180 days [144]. Thus, low-level troponin elevations may reflect ongoing myocardial injury and disease progression, although their role as therapeutic targets remains uncertain. AHF is characterized by in-situ accelerated myocardial necrosis and remodeling in comparison to chronic HF due to increased expression of additional biomarkers of cardiomyocyte injury and myocardial extracellular matrix turnover [144].

### Hypertensive AHF

Hypertensive AHF is characterized by increased afterload and decreased venous capacitance [73, 145, 146]. As a consequence, fluid is redistributed from peripheral vessels and splanchnic vessels into pulmonary circulation. Patients with chronic hypertension who have systolic blood pressure (SBP) > 160 mm Hg experience rapid onset of dyspnea [147].

Hypertension causes LV hypertrophy, stiffness, and diastolic dysfunction, predisposing patients to AHF [44]. It is important to improve forward flow and realign the ventricular–vascular coupling relationship in these patients as simply reducing preload may not be sufficient to achieve this goal. Approximately 20% of AHF patients have SBP > 140 mmHg, consistent with a hypertensive phenotype [147].

### The ventricular–vascular coupling correlation

**Fig. 2:**
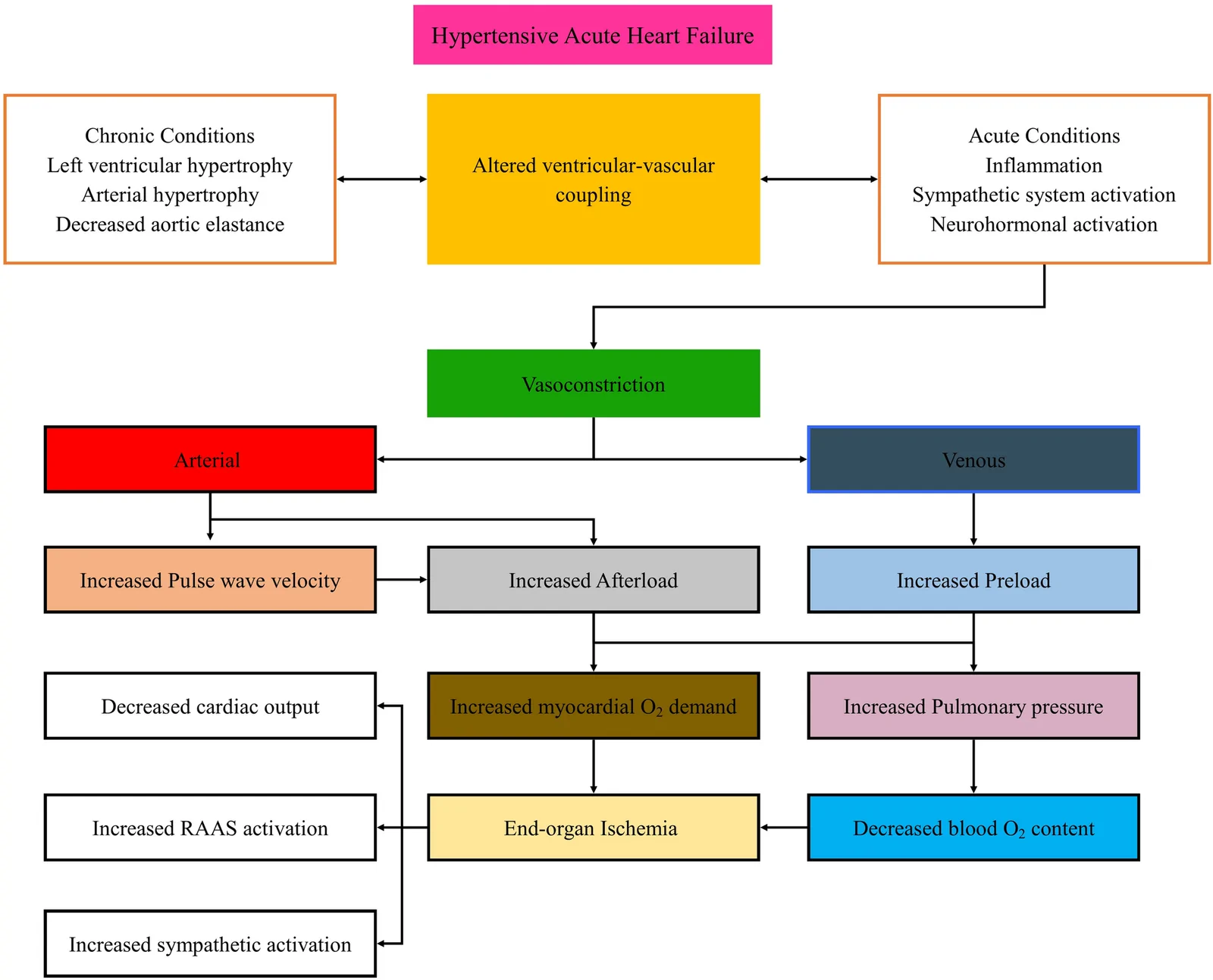
Ventricular-vascular uncoupling and vasoconstriction in hypertensive acute heart failure

Hypertensive AHF is primarily caused by ventricular-vascular uncoupling (Fig. 2), which may be exacerbated by myocardial ischemia, renal dysfunction, volume overload or depletion, and chronic respiratory disorders. The interaction between ventricular and arterial properties is termed ventricular–vascular coupling. The vascular system performs two functions in normal ventricular-vascular interaction: (A) providing blood flow to vital organs and the peripheral circulation, and (B) attenuating high SBP without compromising diastolic flow [148].

Sunagawa et al. [149] proposed an elastic model for the arterial and ventricular systems. In this model, Ea and LV-Ees work in opposition to one another and affect volume changes in slightly different ways. Ventricular-vascular interactions are often described using the Ea/Ees ratio [150]. LV pressure-volume loops can be used to derive these values, where Ees reflects the relationship between end-systolic pressure and volume, whereas Ea represents the effective arterial load. Typically, the Ea/Ees ratio ranges from 0.6 to 1.0, a range generally associated with efficient ventricular–arterial coupling [151].

Both humans with HF and canine models of cardiac disease exhibit ventricular-vascular coupling dysfunction. Imbalance leads to uncoupling, often associated with vascular stiffening. A number of studies have demonstrated vascular stiffening in elderly populations, regardless of cardiovascular disease status [152].

Chronic hypertension is most closely associated with arterial stiffening, which is accelerated and generally accompanied by renal disease and diabetes mellitus [153]. Hypertension results in a progressively greater pulsatile load on the vasculature, resulting in smooth muscle hypertrophy within small-caliber arterioles and inflammation in large elastic arteries. Consequently, LV forward flow is progressively restricted by reduced vascular compliance [153]. A hypertrophic remodeling of the LV maintains the coupling relationship and dissipates transmural stress, altering ventricular geometry and relaxation properties. LV compliance decreases when collagen cross-linking and myocyte protein expression are altered [154].

The Ea/Ees ratio is maintained in the optimal range across widely varying elasticities as the vascular tree becomes increasingly non-compliant. However, elevated Ea and Ees increase hemodynamic instability [150]. A dynamic perturbation, such as exercise or acute stress, increases SBP/afterload and contractility in vivo. Consequently, Ea and Ees are significantly altered acutely. A small increase in preload or afterload in non-compliant states alters Ea and exacerbates SBP or ΔSBP in an exaggerated manner. A normal heart has sufficient cardiac reserve (ΔEes) [155] to cope with heart rate increases, but LV stiffness reduces this response, particularly through preload recruitable stroke work. As a result, patients who suffer from ventricular dysfunction may experience exertional fatigue. In addition to making the system more responsive to loading conditions, LV stiffness makes it less able to accommodate even normal volumes when peripheral resistance or afterload increases suddenly. A sharp increase in LV-EDP and abrupt increase in afterload, causing an altered pressure-volume relationship, is particularly important in hypertensive AHF, as the heart cannot maintain sufficient stroke volume to match vascular resistance [150]. Hypertensive AHF is often associated with HFpEF and HFrEF [156], because both subtypes are sensitive to enhanced afterload.

### Biomolecules

#### Inflammation

Inflammatory cytokines, including tumor necrosis factor (TNF), IL-1β, and IL-6, are elevated in HF, implicating inflammation as a contributing factor. TNF and IL-6 serum levels correlate with HF severity and prognosis. Elevated baseline cytokine levels may even predict future HF development [157]. Chronic inflammation conditions, driven by diabetes, metabolic syndrome, renal failure, hypertension, COPD, and myocyte injury after myocardial infarction or viral infection, may contribute to an increased risk of HF [158]. Growing interest in anti-inflammatory therapeutic strategies has focused attention on inflammation in HF. Biomarkers of inflammation in HF can also identify inappropriate immune responses and guide treatment development [159].

CRP is associated with cardiovascular and non-cardiovascular mortality as an HF biomarker. Elevated high-sensitivity CRP is associated with higher cardiovascular mortality risk regardless of baseline ejection fraction [160]. Non-immunosuppressive treatments such as beta-blockers, RAAS inhibitors, and statins have only modest effects on CRP levels [161]. CRP is an acute-phase protein synthesized by hepatocytes in response to IL-6 signaling, which is induced by IL-1β and TNF [162]. Further therapeutic possibilities are offered by this signal cascade. IL-1β is therefore being investigated as a potential therapeutic target in HF. Both systolic and diastolic function were directly affected by IL-1β injections in animal models [163]. IL-1β has been shown to identify AHF patients at high risk of 1-year mortality [164].

Further research is needed to determine whether biomarker-guided therapy is beneficial, especially given that NT-proBNP and other established biomarkers have not consistently improved outcomes. A preliminary open-label study of Anakinra therapy in seven mice with acute heart failure found an 84% reduction in CRP levels [165]. Anakinra again reduced the inflammatory response in 30 patients with acute heart failure after it was tested in randomized controlled trial (RCT) [166, 167]. NT-proBNP levels, quality-of-life, and peak oxygen consumption were improved in a separate study of 60 patients with acute heart failure treated with Anakinra for 12 weeks after discharge [168, 169]. These results were partially replicated in an RCT of 30 HFpEF patients, in which quality of life, treadmill exercise time, and NT-proBNP levels improved significantly, though oxygen consumption did not [170]. HFpEF is commonly associated with severe obesity, which may account for the negative results in oxygen consumption. A trial of 10,061 patients treated with canakinumab demonstrated significant reductions in HF hospitalizations and HF-related mortality [171, 172].

Elevated proinflammatory cytokines in HF drive sympathetic activation. Targeting TNF-α and soluble TNF receptors may prevent HF deterioration, as these mediators correlate with HF mortality [167]. The recombinant TNF receptor etanercept was used to neutralize TNF-α in chronic HF patients in the RENAISSANCE and RECOVER trials [169]. The RENAISSANCE trial found that patients with heart failure treated with Etanercept had a worse prognosis than those treated with placebo. The ATTACH study, using infliximab for HF, also showed increased HF severity [173].

Pentoxifylline downregulates Fas/APO-1 apoptosis-signaling receptor expression, inhibiting TNF-α and reducing cell death in multiple cell types [157, 174]. There has been a 4-fold reduction in mortality in patients treated with pentoxifylline for heart failure, leading to optimism with regards to this therapy [175]. In vitro and in vivo studies have shown that IVIG influences cytokine production by T cells and monocytes/macrophages [176, 177]. IVIG significantly improved LVEF and reduced NT-proANP in forty patients with chronic heart failure in a placebo-controlled trial [178].

#### Cardiac remodeling

Cardiac remodeling involves cellular and interstitial changes that alter the heart’s size, mass, function, and geometry following acute or chronic injury [179–182]. These mechanisms result in a decrease in cardiac function. Myocardial damage, such as myocardial infarction, usually induces cardiac remodeling [183]. Cellular and interstitial changes can arise from chronic inflammation, subclinical ischemia, and excessive cardiac strain [158].

A metabolic pathway imbalance can also promote remodeling, such as that related to leptin, neprilysin, and aldosterone [184]. Although cardiac remodeling can be quantified using troponin and natriuretic peptides, risk stratification remains challenging because these biomarkers do not fully capture the extent of remodeling. Novel cardiac biomarkers offer promise for refining diagnosis and monitoring treatment.

In most organ systems, growth/differentiation factor-15 (GDF-15) is expressed and regulates inflammation and fibrosis. Additionally, GDF-15 plays an important role in apoptosis. As a result of organ damage, GDF-15 secretion is increased. Cardioprotection is induced by GDF-15 through several cellular signaling pathways, including ALK 1–7 and Smad receptors, epidermal growth factor receptors (EGFR), and NF-kB/JNK/caspase-3 pathways [185]. GDF-15 levels are elevated in both HFrEF and HFpEF and may impact mortality [186]. GDF-15 is an independent predictor of all-cause and cardiovascular mortality in coronary artery disease patients [187]. GDF-15 has also been reported to be predictive of disease entities other than cardiovascular diseases, such as diabetes, malignancies, and chronic kidney disease [188].

Novel cardiac biomarkers sST2 and GDF-15 may provide more refined assessments of cardiac remodeling, reflecting inflammation, cardiac strain, and oxidative stress [189]. They may actually be a strength for prognostic purposes due to their wide range of involvement, initially thought to be a disadvantage.

#### Blood lactate

Blood lactate is a useful metabolic biomarker in AHF, indicating impaired perfusion and organ stress. Elevated lactate is associated with higher high-sensitivity troponin I, aspartate aminotransferase, and endothelin-1 levels, markers of myocardial injury, hepatic dysfunction, and endothelial activation, respectively. Lactate levels were also associated with dynamic changes in NGAL (a renal tubular stress biomarker); NGAL increased during the first 48 h in patients with high baseline lactate but decreased in those with normal levels. Despite similar serum creatinine and rates of worsening renal function, this pattern supports the concept that NGAL reflects early injury before overt dysfunction occurs. Elevated lactate at baseline was also related to a higher 1-year mortality (36% vs. 21%), and remained an independent predictor of poor outcome even after adjustment for other variables (hazard ratio 1.24, 95% CI 1.08–1.41). These findings highlight lactate’s prognostic importance and relationship with multiple organ injury biomarkers, reinforcing its value as an integrative indicator of microcirculatory and systemic stress in AHF [137].

#### Iron deficiency and heart failure

Iron deficiency (ID) is a highly prevalent comorbidity in HF, affecting 37–75% of patients, and independently worsens symptoms, exercise capacity, quality of life, and prognosis regardless of anemia status [190–192]. Current diagnostic criteria distinguish absolute ID (ferritin < 100 µg/L) from functional ID (ferritin 100–299 µg/L with transferrin saturation < 20%) [190, 192]. ESC guidelines recommend intravenous ferric carboxymaltose for symptomatic HFrEF patients with ID, recognizing ID as a therapeutic target [6, 190, 193].

The pathophysiology of ID in HF involves chronic inflammation driven hepcidin upregulation, which blocks intestinal iron absorption by degrading ferroportin, alongside sympathetic overactivity and cardio renal anemia ID interactions [191–193]. ID impairs mitochondrial oxidative phosphorylation and myocardial contractility, contributing to skeletal and cardiac myocyte dysfunction [191, 192]. Oral iron has not shown consistent clinical benefit, whereas intravenous formulations (ferric carboxymaltose, ferric derisomaltose, iron sucrose) bypass these barriers [192, 193]. Major trials (AFFIRM AHF, IRONMAN, HEART FID, FAIR HF, CONFIRM HF) demonstrate that intravenous iron reduces HF hospitalizations, improves quality of life and exercise capacity, with a favorable safety profile [190, 192, 193].

In AHF, intravenous iron therapy remains unsupported by current evidence due to several specific challenges. First, the inflammatory state in AHF biases routine surrogate markers (ferritin and transferrin saturation), limiting their sensitivity and specificity for diagnosing iron deficiency, whereas alternative biomarkers such as soluble transferrin receptor and hepcidin are not routinely available. Second, active infection, present in 10–25% of AHF admissions, represents a controversial contraindication to intravenous iron. Third, clinical trials in the acute setting have so far shown no consistent benefit. The PRACTISE-ASIA-HF trial found no symptomatic or functional improvement despite iron correction, and retrospective data have not demonstrated reduced rehospitalization rates. Trials such as AFFIRM-AHF and FAIR-HF2 are evaluating intravenous iron in stabilized post-AHF patients, but inpatient iron correction is not currently recommended outside well-selected day-hospital protocols [193].

## Conclusion

Acute heart failure is a complex syndrome whose pathophysiology extends far beyond simple hemodynamic derangements. It involves interactions between LV systolic and diastolic dysfunction, neurohormonal activation (particularly RAAS and adrenergic systems), systemic inflammation, endothelial dysfunction, and congestion, both intravascular and venous, that collectively drive multi-organ dysfunction. The ventricular-vascular coupling mismatch, especially in hypertensive AHF, further exacerbates hemodynamic instability. Biomarkers, including inflammatory cytokines, GDF 15, sST2, and blood lactate, may provide prognostic information, while myocardial injury and cardiac remodeling remain central to disease progression. Despite current standard therapies with intravenous diuretics and vasodilators, outcomes remain poor, underscoring the need for individualized treatment strategies guided by congestion, perfusion, blood pressure, clinical phenotype, and underlying pathophysiology. Future research should focus on refining congestion assessment, optimizing decongestive therapies, and exploring novel therapeutic targets, including iron correction in select populations, to improve both short- and long-term outcomes in this vulnerable patient group.

## Data Availability

No datasets were generated or analysed during the current study.

## Abbreviations

ADHF: Acutely decompensated heart failure
AHF: Acute heart failure
ALT: Alanine aminotransferase
ANP: Atrial natriuretic peptide
AR: Adrenergic receptor
AST: Aspartate aminotransferase
BIOSTAT-CHF: The Biology Study to Tailored Treatment in Chronic Heart Failure
BNP: Brain natriuretic peptide
CI: Confidence interval
COPD: Chronic obstructive pulmonary disease
CRP: C reactive protein
CVP: Central venous pressure
Ea: Arterial elastance
EDP: End-diastolic pressure
Ees: End-systolic elastance
EGFR: Epidermal growth factor receptor
eGFR: Estimated glomerular filtration rate
GDF-15: Growth differentiation factor-15
GDMT: Guideline-directed medical therapy
GEDV: Global End-Diastolic Volume
GFR: Glomerular filtration rate
HF: Heart failure
HFmrEF: Heart failure with mid-range ejection fraction
HFpEF: Heart failure with preserved ejection fraction
HFrEF: Heart failure with reduced ejection fraction
HR: Hazard ratio
IAH: Intra-abdominal Hypertension
IAP: Intra-abdominal Pressure
IL: Interleukin
IVIG: Intravenous immunoglobulin
LV: Left ventricle
LVEF: Left ventricular ejection fraction
MR: Mitral regurgitation
NGAL: Neutrophil gelatinase-associated lipocalin
NO: Nitric oxide
NO-cGMP: Nitric oxide-cyclic guanosine monophosphate
NT-proBNP: N-terminal pro-B-type natriuretic peptide
PAOP: Pulmonary Artery Occlusive Pressure
PWV: Pulse wave velocity
RAAS: Renin-angiotensin-aldosterone system
RCT: Randomized controlled trial
RFI: Renal function impairment
ROS: Reactive oxygen species
RVEDV: Right Ventricular End-Diastolic Volume
SBP: Systolic blood pressure
SERCA: Sarcoplasmic reticulum Ca2 + ATPase
SGLT2i: Sodium-glucose cotransporter 2 inhibitors
ST2: Transmembrane receptor 2
SVV: Stroke Volume Variation
Th: T helper
TNF: Tumor necrosis factor
TnI: Troponin I
VEGF-D: Vascular endothelial growth factor-D

## References

[1] Ponikowski P, Voors AA, Anker SD, Bueno H, Cleland JGF, Coats AJS et al (2016) 2016 ESC Guidelines for the diagnosis and treatment of acute and chronic heart failure: The Task Force for the diagnosis and treatment of acute and chronic heart failure of the European Society of Cardiology (ESC)Developed with the special contribution of the Heart Failure Association (HFA) of the ESC. Eur Heart J 37(27):2129–2200. 10.1093/eurheartj/ehw128 27206819

[2] Sotoudeheian M, Hoseini S (2023) Understanding the Pathophysiology of Heart Failure with Mid-Range Ejection Fraction. A Comprehensive Narrative Review

[3] Braunwald E (2013) Heart failure. JACC Heart Fail 1(1):1–20. 10.1016/j.jchf.2012.10.002 24621794

[4] Sotoudeheian MJ, Mirahmadi S-M-S, Pirhayati M, Azarbad R, Nematollahi S, Taghizadeh M et al (2024) Understanding the Role of Galectin-1 in Heart Failure: A Comprehensive Narrative Review. Curr Cardiol Rev 20(1):82–90 10.2174/011573403X274886231227111902 PMC11071677 38192129

[5] Bauersachs J, Soltani S (2023) Heart failure: update of the ESC 2023 guidelines. Herz 10.1007/s00059-023-05221-2 37962569

[6] McDonagh TA, Metra M, Adamo M, Gardner RS, Baumbach A, Böhm M et al (2023) 2023 focused update of the 2021 ESC guidelines for the diagnosis and treatment of acute and chronic heart failure: developed by the task force for the diagnosis and treatment of acute and chronic heart failure of the European Society of Cardiology (ESC) with the special contribution of the Heart Failure Association (HFA) of the ESC. Eur Heart J 44(37):3627–3639 10.1093/eurheartj/ehad195 37622666

[7] Mebazaa A, Yilmaz MB, Levy P, Ponikowski P, Peacock WF, Laribi S et al (2015) Recommendations on pre-hospital & early hospital management of acute heart failure: a consensus paper from the Heart Failure Association of the European Society of Cardiology, the European Society of Emergency Medicine and the Society of Academic Emergency Medicine. Eur J Heart Fail 17(6):544–558. 10.1002/ejhf.289 25999021

[8] Sinnenberg L, Givertz MM (2020) Acute heart failure. Trends Cardiovasc Med 30(2):104–112. 10.1016/j.tcm.2019.03.007 31006522

[9] Ambrosy AP, Fonarow GC, Butler J, Chioncel O, Greene SJ, Vaduganathan M et al (2014) The global health and economic burden of hospitalizations for heart failure: lessons learned from hospitalized heart failure registries. J Am Coll Cardiol 63(12):1123–1133. 10.1016/j.jacc.2013.11.053 24491689

[10] Crespo-Leiro MG, Anker SD, Maggioni AP, Coats AJ, Filippatos G, Ruschitzka F et al (2016) European Society of Cardiology Heart Failure Long-Term Registry (ESC-HF-LT): 1-year follow-up outcomes and differences across regions. Eur J Heart Fail 18(6):613–625. 10.1002/ejhf.566 27324686

[11] Mentz RJ, O’Connor CM (2016) Pathophysiology and clinical evaluation of acute heart failure. Nat Rev Cardiol 13(1):28–35. 10.1038/nrcardio.2015.134 26370473

[12] Ishihara S, Gayat E, Sato N, Arrigo M, Laribi S, Legrand M et al (2016) Similar hemodynamic decongestion with vasodilators and inotropes: systematic review, meta-analysis, and meta-regression of 35 studies on acute heart failure. Clin Res Cardiol 105(12):971–980. 10.1007/s00392-016-1009-6 27314418

[13] Mebazaa A, Parissis J, Porcher R, Gayat E, Nikolaou M, Boas FV et al (2011) Short-term survival by treatment among patients hospitalized with acute heart failure: the global ALARM-HF registry using propensity scoring methods. Intensive Care Med 37(2):290–301. 10.1007/s00134-010-2073-4 21086112

[14] Mebazaa A, Tolppanen H, Mueller C, Lassus J, DiSomma S, Baksyte G et al (2016) Acute heart failure and cardiogenic shock: a multidisciplinary practical guidance. Intensive Care Med 42(2):147–163. 10.1007/s00134-015-4041-5 26370690

[15] Mebazaa A, Combes A, van Diepen S, Hollinger A, Katz JN, Landoni G et al (2018) Management of cardiogenic shock complicating myocardial infarction. Intensive Care Med 44(6):760–773. 10.1007/s00134-018-5214-9 29767322

[16] Follath F, Yilmaz MB, Delgado JF, Parissis JT, Porcher R, Gayat E et al (2011) Clinical presentation, management and outcomes in the Acute Heart Failure Global Survey of Standard Treatment (ALARM-HF). Intensive Care Med 37(4):619–626. 10.1007/s00134-010-2113-0 21210078

[17] Greene SJ, Fonarow GC, Vaduganathan M, Khan SS, Butler J, Gheorghiade M (2015) The vulnerable phase after hospitalization for heart failure. Nat Rev Cardiol 12(4):220–229. 10.1038/nrcardio.2015.14 25666406

[18] Allen LA, O’Connor CM (2007) Management of acute decompensated heart failure. CMAJ 176(6):797–805. 10.1503/cmaj.051620 PMC1808524 17353535

[19] Tromp J, Westenbrink BD, Ouwerkerk W, van Veldhuisen DJ, Samani NJ, Ponikowski P et al (2018) Identifying Pathophysiological Mechanisms in Heart Failure With Reduced Versus Preserved Ejection Fraction. J Am Coll Cardiol 72(10):1081–1090. 10.1016/j.jacc.2018.06.050 30165978

[20] Tromp J, Khan MAF, Mentz RJ, O’Connor CM, Metra M, Dittrich HC et al (2017) Biomarker Profiles of Acute Heart Failure Patients With a Mid-Range Ejection Fraction. JACC Heart Fail 5(7):507–517. 10.1016/j.jchf.2017.04.007 28624483

[21] Arrigo M, Parissis JT, Akiyama E, Mebazaa A (2016) Understanding acute heart failure: pathophysiology and diagnosis. Eur Heart J Supplements 18(supplG):G11–G8. 10.1093/eurheartj/suw044

[22] Arrigo M, Jessup M, Mullens W, Reza N, Shah AM, Sliwa K et al (2020) Acute heart failure. Nat Rev Dis Primers 6(1):16. 10.1038/s41572-020-0151-7 PMC7714436 32139695

[23] Shah AM (2013) Ventricular remodeling in heart failure with preserved ejection fraction. Curr Heart Fail Rep 10(4):341–349. 10.1007/s11897-013-0166-4 PMC4467787 24097113

[24] Wang M, Shah AM (2015) Age-associated pro-inflammatory remodeling and functional phenotype in the heart and large arteries. J Mol Cell Cardiol 83:101–111. 10.1016/j.yjmcc.2015.02.004 PMC4459900 25665458

[25] De Angelis E, Pecoraro M, Rusciano MR, Ciccarelli M, Popolo A (2019) Cross-Talk between Neurohormonal Pathways and the Immune System in Heart Failure: A Review of the Literature. Int J Mol Sci 20(7). 10.3390/ijms20071698 PMC6480265 30959745

[26] Vergaro G, Aimo A, Prontera C, Ghionzoli N, Arzilli C, Zyw L et al (2019) Sympathetic and renin-angiotensin-aldosterone system activation in heart failure with preserved, mid-range and reduced ejection fraction. Int J Cardiol 296:91–97. 10.1016/j.ijcard.2019.08.040 31443984

[27] Yancy CW, Jessup M, Bozkurt B, Butler J, Casey DE Jr., Drazner MH et al (2013) 2013 ACCF/AHA guideline for the management of heart failure: a report of the American College of Cardiology Foundation/American Heart Association Task Force on Practice Guidelines. J Am Coll Cardiol 62(16):e147–239. 10.1016/j.jacc.2013.05.019 23747642

[28] Kovács Á, Barta J (2023) Renin–Angiotensin–Aldosterone System as an Old New Target in Heart Failure Therapy. In: Dhalla NS, Bhullar SK, Shah AK (eds) The Renin Angiotensin System in Cardiovascular Disease. Springer International Publishing, Cham, pp 307–330

[29] Aronson D, Burger AJ (2003) Neurohormonal prediction of mortality following admission for decompensated heart failure. Am J Cardiol 91(2):245–248. 10.1016/s0002-9149(02)03119-3 12521645

[30] Aronson D, Burger AJ (2003) Neurohumoral activation and ventricular arrhythmias in patients with decompensated congestive heart failure: role of endothelin. Pacing Clin Electrophysiol 26(3):703–710. 10.1046/j.1460-9592.2003.00120.x 12698670

[31] Reudelhuber TL, Bernstein KE, Delafontaine P (2007) Is angiotensin II a direct mediator of left ventricular hypertrophy? Time for another look. Hypertension 49(6):1196–1201. 10.1161/hypertensionaha.106.075085 PMC3227117 17452509

[32] Milo-Cotter O, Cotter-Davison B, Lombardi C, Sun H, Bettari L, Bugatti S et al (2011) Neurohormonal activation in acute heart failure: results from VERITAS. Cardiology 119(2):96–105. 10.1159/000330409 21912122

[33] Chin BS, Conway DS, Chung NA, Blann AD, Gibbs CR, Lip GY (2003) Interleukin-6, tissue factor and von Willebrand factor in acute decompensated heart failure: relationship to treatment and prognosis. Blood Coagul Fibrinolysis 14(6):515–521. 10.1097/00001721-200309000-00001 12960603

[34] Kalogeropoulos AP, Tang WH, Hsu A, Felker GM, Hernandez AF, Troughton RW et al (2014) High-sensitivity C-reactive protein in acute heart failure: insights from the ASCEND-HF trial. J Card Fail 20(5):319–326. 10.1016/j.cardfail.2014.02.002 24530944

[35] Ahmad T, Fiuzat M, Felker GM, O’Connor C (2012) Novel biomarkers in chronic heart failure. Nat Rev Cardiol 9(6):347–359. 10.1038/nrcardio.2012.37 22450126

[36] Dmour BA, Costache AD, Dmour A, Huzum B, Duca ȘT, Chetran A et al (2023) Could Endothelin-1 Be a Promising Neurohormonal Biomarker in Acute Heart Failure? Diagnostics (Basel). 13(13). 10.3390/diagnostics13132277 PMC10340204 37443671

[37] Mann DL (2002) Inflammatory mediators and the failing heart: past, present, and the foreseeable future. Circ Res 91(11):988–998. 10.1161/01.res.0000043825.01705.1b 12456484

[38] Girerd N, Pang PS, Swedberg K, Fought A, Kwasny MJ, Subacius H et al (2013) Serum aldosterone is associated with mortality and re-hospitalization in patients with reduced ejection fraction hospitalized for acute heart failure: analysis from the EVEREST trial. Eur J Heart Fail 15(11):1228–1235. 10.1093/eurjhf/hft100 23787720

[39] Goldsmith SR, Burkhoff D, Gustafsson F, Voors A, Zannad F, Kolkhof P et al (2021) Dual Vasopressin Receptor Antagonism to Improve Congestion in Patients With Acute Heart Failure: Design of the AVANTI Trial. J Card Fail 27(2):233–241. 10.1016/j.cardfail.2020.10.007 33188886

[40] Ronco C, Cicoira M, McCullough PA (2012) Cardiorenal syndrome type 1: pathophysiological crosstalk leading to combined heart and kidney dysfunction in the setting of acutely decompensated heart failure. J Am Coll Cardiol 60(12):1031–1042. 10.1016/j.jacc.2012.01.077 22840531

[41] Mentz RJ, Stevens SR, DeVore AD, Lala A, Vader JM, AbouEzzeddine OF et al (2015) Decongestion strategies and renin-angiotensin-aldosterone system activation in acute heart failure. JACC Heart Fail 3(2):97–107. 10.1016/j.jchf.2014.09.003 PMC4324057 25543972

[42] Felker GM, Butler J, Collins SP, Cotter G, Davison BA, Ezekowitz JA et al (2015) Heart failure therapeutics on the basis of a biased ligand of the angiotensin-2 type 1 receptor. Rationale and design of the BLAST-AHF study (Biased Ligand of the Angiotensin Receptor Study in Acute Heart Failure). JACC Heart Fail 3(3):193–201. 10.1016/j.jchf.2014.09.008 25650371

[43] O’Leary DS (2006) Altered reflex cardiovascular control during exercise in heart failure: animal studies. Exp Physiol 91(1):73–77. 10.1113/expphysiol.2005.031179 16179406

[44] Gheorghiade M, De Luca L, Fonarow GC, Filippatos G, Metra M, Francis GS (2005) Pathophysiologic targets in the early phase of acute heart failure syndromes. Am J Cardiol 96(6a):11 g–7g. 10.1016/j.amjcard.2005.07.016 16196154

[45] Colombo PC, Onat D, Sabbah HN (2008) Acute heart failure as acute endothelitis--Interaction of fluid overload and endothelial dysfunction. Eur J Heart Fail 10(2):170–175. 10.1016/j.ejheart.2007.12.007 18279772

[46] Yu CM, Wang L, Chau E, Chan RH, Kong SL, Tang MO et al (2005) Intrathoracic impedance monitoring in patients with heart failure: correlation with fluid status and feasibility of early warning preceding hospitalization. Circulation 112(6):841–848. 10.1161/circulationaha.104.492207 16061743

[47] Yasmin, McEniery CM, Wallace S, Mackenzie IS, Cockcroft JR, Wilkinson IB (2004) C-reactive protein is associated with arterial stiffness in apparently healthy individuals. Arterioscler Thromb Vasc Biol 24(5):969–974. 10.1161/01.ATV.zhq0504.0173 15001456

[48] Viau DM, Sala-Mercado JA, Spranger MD, O’Leary DS, Levy PD (2015) The pathophysiology of hypertensive acute heart failure. Heart 101(23):1861–1867. 10.1136/heartjnl-2015-307461 26123135

[49] Gheorghiade M, Follath F, Ponikowski P, Barsuk JH, Blair JE, Cleland JG et al (2010) Assessing and grading congestion in acute heart failure: a scientific statement from the acute heart failure committee of the heart failure association of the European Society of Cardiology and endorsed by the European Society of Intensive Care Medicine. Eur J Heart Fail 12(5):423–433. 10.1093/eurjhf/hfq045 20354029

[50] Fudim M, Hernandez AF, Felker GM (2017) Role of Volume Redistribution in the Congestion of Heart Failure. J Am Heart Assoc 6(8). 10.1161/jaha.117.006817 PMC5586477 28862947

[51] Braam B, Cupples WA, Joles JA, Gaillard C (2012) Systemic arterial and venous determinants of renal hemodynamics in congestive heart failure. Heart Fail Rev 17(2):161–175. 10.1007/s10741-011-9246-2 21553212

[52] Njoroge JN, Teerlink JR (2021) Pathophysiology and Therapeutic Approaches to Acute Decompensated Heart Failure. Circ Res 128(10):1468–1486. 10.1161/circresaha.121.318186 PMC8126502 33983837

[53] Asgar AW, Mack MJ, Stone GW (2015) Secondary mitral regurgitation in heart failure: pathophysiology, prognosis, and therapeutic considerations. J Am Coll Cardiol 65(12):1231–1248. 10.1016/j.jacc.2015.02.009 25814231

[54] Cork DP, McCullough PA, Mehta HS, Barker CM, Gunnarsson C, Ryan MP et al (2020) Impact of mitral regurgitation on cardiovascular hospitalization and death in newly diagnosed heart failure patients. ESC Heart Fail 7(4):1502–1509. 10.1002/ehf2.12653 PMC7373926 32469120

[55] Kajimoto K, Sato N, Takano T (2016) Functional mitral regurgitation at discharge and outcomes in patients hospitalized for acute decompensated heart failure with a preserved or reduced ejection fraction. Eur J Heart Fail 18(8):1051–1059. 10.1002/ejhf.562 27212582

[56] Kubo S, Kawase Y, Hata R, Maruo T, Tada T, Kadota K (2018) Dynamic severe mitral regurgitation on hospital arrival as prognostic predictor in patients hospitalized for acute decompensated heart failure. Int J Cardiol 273:177–182. 10.1016/j.ijcard.2018.09.093 30274752

[57] Volterrani M, Spoletini I, Angermann C, Rosano G, Coats AJ (2019) Implantable devices for heart failure monitoring: the CardioMEMS™ system. Eur Heart J Suppl 21(Suppl M):M50–m3. 10.1093/eurheartj/suz265 PMC6937499 31908617

[58] Zile MR, Bennett TD, St John Sutton M, Cho YK, Adamson PB, Aaron MF et al (2008) Transition from chronic compensated to acute decompensated heart failure: pathophysiological insights obtained from continuous monitoring of intracardiac pressures. Circulation 118(14):1433–1441. 10.1161/circulationaha.108.783910 18794390

[59] Miller WL, Mullan BP (2014) Understanding the heterogeneity in volume overload and fluid distribution in decompensated heart failure is key to optimal volume management: role for blood volume quantitation. JACC Heart Fail 2(3):298–305. 10.1016/j.jchf.2014.02.007 24952698

[60] Gronda E, Dusi V, D’Elia E, Iacoviello M, Benvenuto E, Vanoli E (2022) Sympathetic activation in heart failure(). Eur Heart J Suppl 24(Suppl E):E4–e11. 10.1093/eurheartjsupp/suac030 PMC9385124 35991621

[61] Fallick C, Sobotka PA, Dunlap ME (2011) Sympathetically mediated changes in capacitance: redistribution of the venous reservoir as a cause of decompensation. Circ Heart Fail 4(5):669–675. 10.1161/circheartfailure.111.961789 21934091

[62] Chaudhry SI, Wang Y, Concato J, Gill TM, Krumholz HM (2007) Patterns of weight change preceding hospitalization for heart failure. Circulation 116(14):1549–1554. 10.1161/circulationaha.107.690768 PMC2892745 17846286

[63] Nijst P, Verbrugge FH, Grieten L, Dupont M, Steels P, Tang WHW et al (2015) The pathophysiological role of interstitial sodium in heart failure. J Am Coll Cardiol 65(4):378–388. 10.1016/j.jacc.2014.11.025 25634838

[64] Titze J, Shakibaei M, Schafflhuber M, Schulze-Tanzil G, Porst M, Schwind KH et al (2004) Glycosaminoglycan polymerization may enable osmotically inactive Na+ storage in the skin. Am J Physiol Heart Circ Physiol 287(1):H203–H208. 10.1152/ajpheart.01237.2003 14975935

[65] Cenaj O, Allison DHR, Imam R, Zeck B, Drohan LM, Chiriboga L et al (2021) Evidence for continuity of interstitial spaces across tissue and organ boundaries in humans. Commun Biol 4(1):436. 10.1038/s42003-021-01962-0 PMC8012658 33790388

[66] Hartupee J, Mann DL (2017) Neurohormonal activation in heart failure with reduced ejection fraction. Nat Rev Cardiol 14(1):30–38. 10.1038/nrcardio.2016.163 PMC5286912 27708278

[67] Mullens W, Verbrugge FH, Nijst P, Tang WHW (2017) Renal sodium avidity in heart failure: from pathophysiology to treatment strategies. Eur Heart J 38(24):1872–1882. 10.1093/eurheartj/ehx035 28329085

[68] McKie PM, Schirger JA, Costello-Boerrigter LC, Benike SL, Harstad LK, Bailey KR et al (2011) Impaired natriuretic and renal endocrine response to acute volume expansion in pre-clinical systolic and diastolic dysfunction. J Am Coll Cardiol 58(20):2095–2103. 10.1016/j.jacc.2011.07.042 PMC3835631 22051332

[69] Mullens W, Tang WH (2011) The early intertwining of the heart and the kidney through an impaired natriuretic response to acute volume expansion. J Am Coll Cardiol 58(20):2104–2105. 10.1016/j.jacc.2011.05.064 22051333

[70] Mullens W, Abrahams Z, Francis GS, Sokos G, Taylor DO, Starling RC et al (2009) Importance of venous congestion for worsening of renal function in advanced decompensated heart failure. J Am Coll Cardiol 53(7):589–596. 10.1016/j.jacc.2008.05.068 PMC2856960 19215833

[71] Damman K, van Deursen VM, Navis G, Voors AA, van Veldhuisen DJ, Hillege HL (2009) Increased central venous pressure is associated with impaired renal function and mortality in a broad spectrum of patients with cardiovascular disease. J Am Coll Cardiol 53(7):582–588. 10.1016/j.jacc.2008.08.080 19215832

[72] Nijst P, Martens P, Dupont M, Tang WHW, Mullens W (2017) Intrarenal Flow Alterations During Transition From Euvolemia to Intravascular Volume Expansion in Heart Failure Patients. JACC Heart Fail 5(9):672–681. 10.1016/j.jchf.2017.05.006 28711449

[73] Cotter G, Metra M, Milo-Cotter O, Dittrich HC, Gheorghiade M (2008) Fluid overload in acute heart failure–re-distribution and other mechanisms beyond fluid accumulation. Eur J Heart Fail 10(2):165–169. 10.1016/j.ejheart.2008.01.007 18279771

[74] Fudim M, Khan MS, Paracha AA, Sunagawa K, Burkhoff D (2022) Targeting Preload in Heart Failure: Splanchnic Nerve Blockade and Beyond. Circ Heart Fail 15(3):e009340. 10.1161/circheartfailure.121.009340 PMC8931843 35290092

[75] Verbrugge FH, Dupont M, Steels P, Grieten L, Malbrain M, Tang WH et al (2013) Abdominal contributions to cardiorenal dysfunction in congestive heart failure. J Am Coll Cardiol 62(6):485–495. 10.1016/j.jacc.2013.04.070 23747781

[76] Cheatham ML (2009) Abdominal compartment syndrome: pathophysiology and definitions. Scand J Trauma Resusc Emerg Med 17(1):10 10.1186/1757-7241-17-10 PMC2654860 19254364

[77] Łagosz P, Sokolski M, Biegus J, Tycinska A, Zymlinski R (2022) Elevated intra-abdominal pressure: A review of current knowledge. World J Clin cases 10(10):3005 10.12998/wjcc.v10.i10.3005 PMC9082714 35647129

[78] Fonarow GC, Abraham WT, Albert NM, Stough WG, Gheorghiade M, Greenberg BH et al (2008) Factors identified as precipitating hospital admissions for heart failure and clinical outcomes: findings from OPTIMIZE-HF. Arch Intern Med 168(8):847–854. 10.1001/archinte.168.8.847 18443260

[79] Arrigo M, Gayat E, Parenica J, Ishihara S, Zhang J, Choi DJ et al (2017) Precipitating factors and 90-day outcome of acute heart failure: a report from the intercontinental GREAT registry. Eur J Heart Fail 19(2):201–208. 10.1002/ejhf.682 27790819

[80] Arrigo M, Tolppanen H, Sadoune M, Feliot E, Teixeira A, Laribi S et al (2016) Effect of precipitating factors of acute heart failure on readmission and long-term mortality. ESC Heart Fail 3(2):115–121. 10.1002/ehf2.12083 PMC5066631 27812386

[81] Platz E, Jhund PS, Claggett BL, Pfeffer MA, Swedberg K, Granger CB et al (2018) Prevalence and prognostic importance of precipitating factors leading to heart failure hospitalization: recurrent hospitalizations and mortality. Eur J Heart Fail 20(2):295–303. 10.1002/ejhf.901 PMC5826811 28872259

[82] Miró Ò, Takagi K, Gayat É, Gil V, Llorens P, Martín-Sánchez FJ et al (2020) Time-pattern of adverse outcomes after an infection-triggered acute heart failure decompensation and the influence of early antibiotic administration and hospitalisation: results of the PAPRICA-3 study. Clin Res Cardiol 109(1):34–45. 10.1007/s00392-019-01481-3 31037410

[83] Schiff GD, Fung S, Speroff T, McNutt RA (2003) Decompensated heart failure: symptoms, patterns of onset, and contributing factors. Am J Med 114(8):625–630. 10.1016/s0002-9343(03)00132-3 12798449

[84] Burchell AE, Sobotka PA, Hart EC, Nightingale AK, Dunlap ME (2013) Chemohypersensitivity and autonomic modulation of venous capacitance in the pathophysiology of acute decompensated heart failure. Curr Heart Fail Rep 10(2):139–146. 10.1007/s11897-013-0135-y 23504401

[85] Nohria A, Hasselblad V, Stebbins A, Pauly DF, Fonarow GC, Shah M et al (2008) Cardiorenal interactions: insights from the ESCAPE trial. J Am Coll Cardiol 51(13):1268–1274. 10.1016/j.jacc.2007.08.072 18371557

[86] Ajami M, Davoodi SH, Habibey R, Namazi N, Soleimani M, Pazoki-Toroudi H (2013) Effect of DHA + EPA on oxidative stress and apoptosis induced by ischemia‐reperfusion in rat kidneys. Fundam Clin Pharmacol 27(6):593–602 10.1111/j.1472-8206.2012.01066.x 22943605

[87] Javedan G, Shidfar F, Davoodi SH, Ajami M, Gorjipour F, Sureda A et al (2016) Conjugated linoleic acid rat pretreatment reduces renal damage in ischemia/reperfusion injury: Unraveling antiapoptotic mechanisms and regulation of phosphorylated mammalian target of rapamycin. Mol Nutr Food Res 60(12):2665–2677 10.1002/mnfr.201600112 27466783

[88] ter Maaten JM, Valente MA, Damman K, Hillege HL, Navis G, Voors AA (2015) Diuretic response in acute heart failure-pathophysiology, evaluation, and therapy. Nat Rev Cardiol 12(3):184–192. 10.1038/nrcardio.2014.215 25560378

[89] Ronco C, Haapio M, House AA, Anavekar N, Bellomo R (2008) Cardiorenal syndrome. J Am Coll Cardiol 52(19):1527–1539. 10.1016/j.jacc.2008.07.051 19007588

[90] Mentz RJ, Lewis EF (2011) Epidemiology of cardiorenal syndrome. Cardiol Clin 29(2):301–314. 10.1016/j.ccl.2011.03.004 21459251

[91] Pazoki-Toroudi HR, Hesami A, Vahidi S, Sahebjam F, Seifi B, Djahanguiri B (2003) The preventive effect of captopril or enalapril on reperfusion injury of the kidney of rats is independent of angiotensin II AT1 receptors. Fundam Clin Pharmacol 17(5):595–598 10.1046/j.1472-8206.2003.00188.x 14703720

[92] Colombo PC, Jorde UP (2010) The active role of venous congestion in the pathophysiology of acute decompensated heart failure. Rev Esp Cardiol 63(1):5–8. 10.1016/s1885-5857(10)70002-5 20089219

[93] Gutiérrez E, Flammer AJ, Lerman LO, Elízaga J, Lerman A, Fernández-Avilés F (2013) Endothelial dysfunction over the course of coronary artery disease. Eur Heart J 34(41):3175–3181. 10.1093/eurheartj/eht351 PMC3814514 24014385

[94] Marti CN, Gheorghiade M, Kalogeropoulos AP, Georgiopoulou VV, Quyyumi AA, Butler J (2012) Endothelial dysfunction, arterial stiffness, and heart failure. J Am Coll Cardiol 60(16):1455–1469. 10.1016/j.jacc.2011.11.082 22999723

[95] Sotoudeheian MJ, Azarbad R, Pirhayati M, Moradi M, Pazoki-Toroudi H Targeting SARS-CoV-2-Induced Cardiovascular Injury: Exploring the Potential of Ponatinib in Mitigating Cardiovascular Necroptosis in COVID-19. Current pharmaceutical biotechnology 10.2174/0113892010324744240916110446 39328138

[96] Sotoudeheian M, Soleimani M, Farahmandian N (2023) Molecular Pathways Disturbances during COVID-19 Lead to Cardiomyocyte Necroptosis

[97] Colombo PC, Onat D, Harxhi A, Demmer RT, Hayashi Y, Jelic S et al (2014) Peripheral venous congestion causes inflammation, neurohormonal, and endothelial cell activation. Eur Heart J 35(7):448–454. 10.1093/eurheartj/eht456 PMC3924182 24265434

[98] Premer C, Kanelidis AJ, Hare JM, Schulman IH (2019) Rethinking Endothelial Dysfunction as a Crucial Target in Fighting Heart Failure. Mayo Clin Proc Innov Qual Outcomes 3(1):1–13. 10.1016/j.mayocpiqo.2018.12.006 PMC6408687 30899903

[99] Zuchi C, Tritto I, Carluccio E, Mattei C, Cattadori G, Ambrosio G (2020) Role of endothelial dysfunction in heart failure. Heart Fail Rev 25(1):21–30. 10.1007/s10741-019-09881-3 31686283

[100] Parrinello G, Greene SJ, Torres D, Alderman M, Bonventre JV, Di Pasquale P et al (2015) Water and sodium in heart failure: a spotlight on congestion. Heart Fail Rev 20(1):13–24. 10.1007/s10741-014-9438-7 PMC4405162 24942806

[101] Volpe M, Carnovali M, Mastromarino V (2016) The natriuretic peptides system in the pathophysiology of heart failure: from molecular basis to treatment. Clin Sci (Lond) 130(2):57–77. 10.1042/cs20150469 PMC5233571 26637405

[102] MacIver DH, Adeniran I, MacIver IR, Revell A, Zhang H (2016) Physiological mechanisms of pulmonary hypertension. Am Heart J 180:1–11. 10.1016/j.ahj.2016.07.003 27659877

[103] Borné Y, Gränsbo K, Nilsson J, Melander O, Orho-Melander M, Smith JG et al (2018) Vascular Endothelial Growth Factor D, Pulmonary Congestion, and Incidence of Heart Failure. J Am Coll Cardiol 71(5):580–582. 10.1016/j.jacc.2017.11.058 29406866

[104] Houston BA, Tedford RJ, Baxley RL, Sykes B, Powers ER, Nielsen CD et al (2019) Relation of Lymphangiogenic Factor Vascular Endothelial Growth Factor-D to Elevated Pulmonary Artery Wedge Pressure. Am J Cardiol 124(5):756–762. 10.1016/j.amjcard.2019.05.056 31296367

[105] von Moos S, Segerer S, Davenport A, Sadoune M, Gerritsen K, Pottecher J et al (2021) Vascular endothelial growth factor D is a biomarker of fluid overload in haemodialysis patients. Nephrol Dial Transpl 36(3):529–536. 10.1093/ndt/gfz281 31923307

[106] Ware LB, Matthay MA (2005) Clinical practice. Acute pulmonary edema. N Engl J Med 353(26):2788–2796. 10.1056/NEJMcp052699 16382065

[107] Harjola VP, Mullens W, Banaszewski M, Bauersachs J, Brunner-La Rocca HP, Chioncel O et al (2017) Organ dysfunction, injury and failure in acute heart failure: from pathophysiology to diagnosis and management. A review on behalf of the Acute Heart Failure Committee of the Heart Failure Association (HFA) of the European Society of Cardiology (ESC). Eur J Heart Fail 19(7):821–836. 10.1002/ejhf.872 PMC5734941 28560717

[108] Tanwar P, Naagar M, Malik G, Alam M, Singh T, Singh et al (2022) Relationship between Right Heart Failure and Cardio-Renal Syndrome: A review. World J Biology Pharm Health Sci 13:122–137. 10.30574/wjbphs.2023.13.1.0011

[109] Poelzl G, Ess M, Mussner-Seeber C, Pachinger O, Frick M, Ulmer H (2012) Liver dysfunction in chronic heart failure: prevalence, characteristics and prognostic significance. Eur J Clin Invest 42(2):153–163. 10.1111/j.1365-2362.2011.02573.x 21806605

[110] Nikolaou M, Parissis J, Yilmaz MB, Seronde MF, Kivikko M, Laribi S et al (2013) Liver function abnormalities, clinical profile, and outcome in acute decompensated heart failure. Eur Heart J 34(10):742–749. 10.1093/eurheartj/ehs332 23091203

[111] Ebert EC (2006) Hypoxic liver injury. Mayo Clin Proc 81(9):1232–1236. 10.4065/81.9.1232 16970220

[112] Henrion J, Schapira M, Luwaert R, Colin L, Delannoy A, Heller FR (2003) Hypoxic hepatitis: clinical and hemodynamic study in 142 consecutive cases. Med (Baltim) 82(6):392–406. 10.1097/01.md.0000101573.54295.bd 14663289

[113] Valentova M, von Haehling S, Bauditz J, Doehner W, Ebner N, Bekfani T et al (2016) Intestinal congestion and right ventricular dysfunction: a link with appetite loss, inflammation, and cachexia in chronic heart failure. Eur Heart J 37(21):1684–1691. 10.1093/eurheartj/ehw008 26865478

[114] Hillege HL, Nitsch D, Pfeffer MA, Swedberg K, McMurray JJ, Yusuf S et al (2006) Renal function as a predictor of outcome in a broad spectrum of patients with heart failure. Circulation 113(5):671–678. 10.1161/circulationaha.105.580506 16461840

[115] Dries DL, Exner DV, Domanski MJ, Greenberg B, Stevenson LW (2000) The prognostic implications of renal insufficiency in asymptomatic and symptomatic patients with left ventricular systolic dysfunction. J Am Coll Cardiol 35(3):681–689. 10.1016/s0735-1097(99)00608-7 10716471

[116] Heywood JT, Fonarow GC, Costanzo MR, Mathur VS, Wigneswaran JR, Wynne J (2007) High prevalence of renal dysfunction and its impact on outcome in 118,465 patients hospitalized with acute decompensated heart failure: a report from the ADHERE database. J Card Fail 13(6):422–430. 10.1016/j.cardfail.2007.03.011 17675055

[117] Damman K, Navis G, Voors AA, Asselbergs FW, Smilde TD, Cleland JG et al (2007) Worsening renal function and prognosis in heart failure: systematic review and meta-analysis. J Card Fail 13(8):599–608. 10.1016/j.cardfail.2007.04.008 17923350

[118] Owan TE, Hodge DO, Herges RM, Jacobsen SJ, Roger VL, Redfield MM (2006) Secular trends in renal dysfunction and outcomes in hospitalized heart failure patients. J Card Fail 12(4):257–262. 10.1016/j.cardfail.2006.02.007 16679257

[119] Smith GL, Vaccarino V, Kosiborod M, Lichtman JH, Cheng S, Watnick SG et al (2003) Worsening renal function: what is a clinically meaningful change in creatinine during hospitalization with heart failure? J Card Fail 9(1):13–25. 10.1054/jcaf.2003.3 12612868

[120] Forman DE, Butler J, Wang Y, Abraham WT, O’Connor CM, Gottlieb SS et al (2004) Incidence, predictors at admission, and impact of worsening renal function among patients hospitalized with heart failure. J Am Coll Cardiol 43(1):61–67. 10.1016/j.jacc.2003.07.031 14715185

[121] Aronson D, Burger AJ (2010) The relationship between transient and persistent worsening renal function and mortality in patients with acute decompensated heart failure. J Card Fail 16(7):541–547. 10.1016/j.cardfail.2010.02.001 20610229

[122] Testani JM, Chen J, McCauley BD, Kimmel SE, Shannon RP (2010) Potential effects of aggressive decongestion during the treatment of decompensated heart failure on renal function and survival. Circulation 122(3):265–272. 10.1161/circulationaha.109.933275 PMC3025294 20606118

[123] Kazory A, Elkayam U (2014) Cardiorenal interactions in acute decompensated heart failure: contemporary concepts facing emerging controversies. J Card Fail 20(12):1004–1011. 10.1016/j.cardfail.2014.09.005 25230240

[124] Aronson D, Abassi Z, Allon E, Burger AJ (2013) Fluid loss, venous congestion, and worsening renal function in acute decompensated heart failure. Eur J Heart Fail 15(6):637–643. 10.1093/eurjhf/hft036 23475780

[125] Legrand M, Mebazaa A, Ronco C, Januzzi JL (2014) Jr. When cardiac failure, kidney dysfunction, and kidney injury intersect in acute conditions: the case of cardiorenal syndrome. Crit Care Med 42(9):2109–2117. 10.1097/ccm.0000000000000404 24810531

[126] Mullens W, Abrahams Z, Skouri HN, Francis GS, Taylor DO, Starling RC et al (2008) Elevated intra-abdominal pressure in acute decompensated heart failure: a potential contributor to worsening renal function? J Am Coll Cardiol 51(3):300–306. 10.1016/j.jacc.2007.09.043 18206740

[127] Ahmad T, Jackson K, Rao VS, Tang WHW, Brisco-Bacik MA, Chen HH et al (2018) Worsening Renal Function in Patients With Acute Heart Failure Undergoing Aggressive Diuresis Is Not Associated With Tubular Injury. Circulation 137(19):2016–2028. 10.1161/circulationaha.117.030112 PMC6066176 29352071

[128] Maisel AS, Wettersten N, van Veldhuisen DJ, Mueller C, Filippatos G, Nowak R et al (2016) Neutrophil Gelatinase-Associated Lipocalin for Acute Kidney Injury During Acute Heart Failure Hospitalizations: The AKINESIS Study. J Am Coll Cardiol 68(13):1420–1431. 10.1016/j.jacc.2016.06.055 27659464

[129] Mullens W, Damman K, Harjola VP, Mebazaa A, Brunner-La Rocca HP, Martens P et al (2019) The use of diuretics in heart failure with congestion - a position statement from the Heart Failure Association of the European Society of Cardiology. Eur J Heart Fail 21(2):137–155. 10.1002/ejhf.1369 30600580

[130] Metra M, Davison B, Bettari L, Sun H, Edwards C, Lazzarini V et al (2012) Is worsening renal function an ominous prognostic sign in patients with acute heart failure? The role of congestion and its interaction with renal function. Circ Heart Fail 5(1):54–62. 10.1161/circheartfailure.111.963413 22167320

[131] Auer J (2013) What does the liver tell us about the failing heart? Eur Heart J 34(10):711–714. 10.1093/eurheartj/ehs440 23257947

[132] Møller S, Bernardi M (2013) Interactions of the heart and the liver. Eur Heart J 34(36):2804–2811. 10.1093/eurheartj/eht246 23853073

[133] Samsky MD, Patel CB, DeWald TA, Smith AD, Felker GM, Rogers JG et al (2013) Cardiohepatic interactions in heart failure: an overview and clinical implications. J Am Coll Cardiol 61(24):2397–2405. 10.1016/j.jacc.2013.03.042 23603231

[134] Rogler G, Rosano G (2014) The heart and the gut. Eur Heart J 35(7):426–430. 10.1093/eurheartj/eht271 23864132

[135] Sandek A, Bauditz J, Swidsinski A, Buhner S, Weber-Eibel J, von Haehling S et al (2007) Altered intestinal function in patients with chronic heart failure. J Am Coll Cardiol 50(16):1561–1569. 10.1016/j.jacc.2007.07.016 17936155

[136] Colombo PC, Doran AC, Onat D, Wong KY, Ahmad M, Sabbah HN et al (2015) Venous congestion, endothelial and neurohormonal activation in acute decompensated heart failure: cause or effect? Curr Heart Fail Rep 12(3):215–222. 10.1007/s11897-015-0254-8 PMC4426008 25740404

[137] Zymliński R, Biegus J, Sokolski M, Siwołowski P, Nawrocka-Millward S, Todd J et al (2018) Increased blood lactate is prevalent and identifies poor prognosis in patients with acute heart failure without overt peripheral hypoperfusion. Eur J Heart Fail 20(6):1011–1018 10.1002/ejhf.1156 29431284

[138] Mullens W, Damman K, Testani JM, Martens P, Mueller C, Lassus J et al (2020) Evaluation of kidney function throughout the heart failure trajectory - a position statement from the Heart Failure Association of the European Society of Cardiology. Eur J Heart Fail 22(4):584–603. 10.1002/ejhf.1697 31908120

[139] Zymliński R, Sokolski M, Biegus J, Siwołowski P, Nawrocka-Millward S, Sokolska JM et al (2019) Multi-organ dysfunction/injury on admission identifies acute heart failure patients at high risk of poor outcome. Eur J Heart Fail 21(6):744–750 10.1002/ejhf.1378 30561066

[140] Kociol RD, Pang PS, Gheorghiade M, Fonarow GC, O’Connor CM, Felker GM (2010) Troponin elevation in heart failure prevalence, mechanisms, and clinical implications. J Am Coll Cardiol 56(14):1071–1078. 10.1016/j.jacc.2010.06.016 20863950

[141] O’Connor CM, Fiuzat M, Lombardi C, Fujita K, Jia G, Davison BA et al (2011) Impact of serial troponin release on outcomes in patients with acute heart failure: analysis from the PROTECT pilot study. Circ Heart Fail 4(6):724–732. 10.1161/circheartfailure.111.961581 21900185

[142] De Peacock WFt T, Fonarow GC, Diercks D, Wynne J, Apple FS et al (2008) Cardiac troponin and outcome in acute heart failure. N Engl J Med 358(20):2117–2126. 10.1056/NEJMoa0706824 18480204

[143] Felker GM, Hasselblad V, Tang WH, Hernandez AF, Armstrong PW, Fonarow GC et al (2012) Troponin I in acute decompensated heart failure: insights from the ASCEND-HF study. Eur J Heart Fail 14(11):1257–1264. 10.1093/eurjhf/hfs110 22764184

[144] Metra M, Cotter G, Davison BA, Felker GM, Filippatos G, Greenberg BH et al (2013) Effect of serelaxin on cardiac, renal, and hepatic biomarkers in the Relaxin in Acute Heart Failure (RELAX-AHF) development program: correlation with outcomes. J Am Coll Cardiol 61(2):196–206. 10.1016/j.jacc.2012.11.005 23273292

[145] Peacock WF, Chandra A, Char D, Collins S, Der Sahakian G, Ding L et al (2014) Clevidipine in acute heart failure: Results of the A Study of Blood Pressure Control in Acute Heart Failure-A Pilot Study (PRONTO). Am Heart J 167(4):529–536. 10.1016/j.ahj.2013.12.023 24655702

[146] Levy P, Compton S, Welch R, Delgado G, Jennett A, Penugonda N et al (2007) Treatment of severe decompensated heart failure with high-dose intravenous nitroglycerin: a feasibility and outcome analysis. Ann Emerg Med 50(2):144–152. 10.1016/j.annemergmed.2007.02.022 17509731

[147] Adams KF Jr., Fonarow GC, Emerman CL, LeJemtel TH, Costanzo MR, Abraham WT et al (2005) Characteristics and outcomes of patients hospitalized for heart failure in the United States: rationale, design, and preliminary observations from the first 100,000 cases in the Acute Decompensated Heart Failure National Registry (ADHERE). Am Heart J 149(2):209–216. 10.1016/j.ahj.2004.08.005 15846257

[148] Ooi H, Chung W, Biolo A (2008) Arterial stiffness and vascular load in heart failure. Congest Heart Fail 14(1):31–36. 10.1111/j.1751-7133.2008.07210.x 18256567

[149] Sunagawa K, Maughan WL, Burkhoff D, Sagawa K (1983) Left ventricular interaction with arterial load studied in isolated canine ventricle. Am J Physiol 245(5 Pt 1):H773–H780. 10.1152/ajpheart.1983.245.5.H773 6638199

[150] Borlaug BA, Kass DA (2008) Ventricular-vascular interaction in heart failure. Heart Fail Clin 4(1):23–36. 10.1016/j.hfc.2007.10.001 PMC2586173 18313622

[151] Starling MR (1993) Left ventricular-arterial coupling relations in the normal human heart. Am Heart J 125(6):1659–1666. 10.1016/0002-8703(93)90756-y 8498308

[152] Redfield MM, Jacobsen SJ, Borlaug BA, Rodeheffer RJ, Kass DA (2005) Age- and gender-related ventricular-vascular stiffening: a community-based study. Circulation 112(15):2254–2262. 10.1161/circulationaha.105.541078 16203909

[153] O’Rourke MF (2007) Arterial aging: pathophysiological principles. Vasc Med 12(4):329–341. 10.1177/1358863x07083392 18048471

[154] Lorell BH, Carabello BA (2000) Left ventricular hypertrophy: pathogenesis, detection, and prognosis. Circulation 102(4):470–479. 10.1161/01.cir.102.4.470 10908222

[155] Fox JM, Maurer MS (2005) Ventriculovascular coupling in systolic and diastolic heart failure. Curr Heart Fail Rep 2(4):204–211. 10.1007/bf02696651 16332314

[156] Schwartzenberg S, Redfield MM, From AM, Sorajja P, Nishimura RA, Borlaug BA (2012) Effects of vasodilation in heart failure with preserved or reduced ejection fraction implications of distinct pathophysiologies on response to therapy. J Am Coll Cardiol 59(5):442–451. 10.1016/j.jacc.2011.09.062 22281246

[157] Deswal A, Petersen NJ, Feldman AM, Young JB, White BG, Mann DL (2001) Cytokines and cytokine receptors in advanced heart failure: an analysis of the cytokine database from the Vesnarinone trial (VEST). Circulation 103(16):2055–2059. 10.1161/01.cir.103.16.2055 11319194

[158] Savarese G, Lund LH (2017) Global Public Health Burden of Heart Failure. Card Fail Rev 3(1):7–11. 10.15420/cfr.2016:25:2 PMC5494150 28785469

[159] Dutka M, Bobiński R, Ulman-Włodarz I, Hajduga M, Bujok J, Pająk C et al (2020) Various aspects of inflammation in heart failure. Heart Fail Rev 25(3):537–548. 10.1007/s10741-019-09875-1 PMC7181445 31705352

[160] Pellicori P, Zhang J, Cuthbert J, Urbinati A, Shah P, Kazmi S et al (2020) High-sensitivity C-reactive protein in chronic heart failure: patient characteristics, phenotypes, and mode of death. Cardiovasc Res 116(1):91–100. 10.1093/cvr/cvz198 31350553

[161] Ikonomidis I, Lekakis JP, Nikolaou M, Paraskevaidis I, Andreadou I, Kaplanoglou T et al (2008) Inhibition of interleukin-1 by anakinra improves vascular and left ventricular function in patients with rheumatoid arthritis. Circulation 117(20):2662–2669. 10.1161/circulationaha.107.731877 18474811

[162] Kalogeropoulos A, Georgiopoulou V, Psaty BM, Rodondi N, Smith AL, Harrison DG et al (2010) Inflammatory markers and incident heart failure risk in older adults: the Health ABC (Health, Aging, and Body Composition) study. J Am Coll Cardiol 55(19):2129–2137. 10.1016/j.jacc.2009.12.045 PMC3267799 20447537

[163] Szekely Y, Arbel Y (2018) A Review of Interleukin-1 in Heart Disease: Where Do We Stand Today? Cardiol Ther 7(1):25–44. 10.1007/s40119-018-0104-3 PMC5986669 29417406

[164] Aleksova A, Beltrami AP, Carriere C, Barbati G, Lesizza P, Perrieri-Montanino M et al (2017) Interleukin-1β levels predict long-term mortality and need for heart transplantation in ambulatory patients affected by idiopathic dilated cardiomyopathy. Oncotarget 8(15):25131–25140. 10.18632/oncotarget.15349 PMC5421915 28212578

[165] Van Tassell BW, Seropian IM, Toldo S, Mezzaroma E, Abbate A (2013) Interleukin-1β induces a reversible cardiomyopathy in the mouse. Inflamm Res 62(7):637–640. 10.1007/s00011-013-0625-0 23649041

[166] Radin MJ, Holycross BJ, Dumitrescu C, Kelley R, Altschuld RA (2008) Leptin modulates the negative inotropic effect of interleukin-1beta in cardiac myocytes. Mol Cell Biochem 315(1–2):179–184. 10.1007/s11010-008-9805-6 18535786

[167] Van Tassell BW, Abouzaki NA, Oddi Erdle C, Carbone S, Trankle CR, Melchior RD et al (2016) Interleukin-1 Blockade in Acute Decompensated Heart Failure: A Randomized, Double-Blinded, Placebo-Controlled Pilot Study. J Cardiovasc Pharmacol 67(6):544–551. 10.1097/fjc.0000000000000378 PMC5749643 26906034

[168] Pascual-Figal DA, Bayes-Genis A, Asensio-Lopez MC, Hernández-Vicente A, Garrido-Bravo I, Pastor-Perez F et al (2019) The Interleukin-1 Axis and Risk of Death in Patients With Acutely Decompensated Heart Failure. J Am Coll Cardiol 73(9):1016–1025. 10.1016/j.jacc.2018.11.054 30846095

[169] Van Tassell BW, Canada J, Carbone S, Trankle C, Buckley L, Oddi Erdle C et al (2017) Interleukin-1 Blockade in Recently Decompensated Systolic Heart Failure: Results From REDHART (Recently Decompensated Heart Failure Anakinra Response Trial). Circ Heart Fail 10(11). 10.1161/circheartfailure.117.004373 PMC5699505 29141858

[170] Felker GM, Anstrom KJ, Adams KF, Ezekowitz JA, Fiuzat M, Houston-Miller N et al (2017) Effect of Natriuretic Peptide-Guided Therapy on Hospitalization or Cardiovascular Mortality in High-Risk Patients With Heart Failure and Reduced Ejection Fraction: A Randomized Clinical Trial. JAMA 318(8):713–720. 10.1001/jama.2017.10565 PMC5605776 28829876

[171] Trankle CR, Canada JM, Cei L, Abouzaki N, Oddi-Erdle C, Kadariya D et al (2018) Usefulness of canakinumab to improve exercise capacity in patients with long-term systolic heart failure and elevated C-reactive protein. Am J Cardiol 122(8):1366–1370 10.1016/j.amjcard.2018.07.002 PMC6522244 30244844

[172] Everett BM, Cornel JH, Lainscak M, Anker SD, Abbate A, Thuren T et al (2019) Anti-inflammatory therapy with canakinumab for the prevention of hospitalization for heart failure. Circulation 139(10):1289–1299 10.1161/CIRCULATIONAHA.118.038010 30586730

[173] Van Tassell BW, Buckley LF, Carbone S, Trankle CR, Canada JM, Dixon DL et al (2017) Interleukin-1 blockade in heart failure with preserved ejection fraction: rationale and design of the Diastolic Heart Failure Anakinra Response Trial 2 (D-HART2). Clin Cardiol 40(9):626–632. 10.1002/clc.22719 PMC5744484 28475816

[174] Topf A, Mirna M, Ohnewein B, Jirak P, Kopp K, Fejzic D et al (2020) The diagnostic and therapeutic value of multimarker analysis in heart failure. An approach to biomarker-targeted therapy. Front Cardiovasc Med 7:579567 10.3389/fcvm.2020.579567 PMC7746655 33344515

[175] Sun M, Dawood F, Wen WH, Chen M, Dixon I, Kirshenbaum LA et al (2004) Excessive tumor necrosis factor activation after infarction contributes to susceptibility of myocardial rupture and left ventricular dysfunction. Circulation 110(20):3221–3228. 10.1161/01.Cir.0000147233.10318.23 15533863

[176] Mann DL, McMurray JJ, Packer M, Swedberg K, Borer JS, Colucci WS et al (2004) Targeted anticytokine therapy in patients with chronic heart failure: results of the Randomized Etanercept Worldwide Evaluation (RENEWAL). Circulation 109(13):1594–1602. 10.1161/01.Cir.0000124490.27666.B2 15023878

[177] Chung ES, Packer M, Lo KH, Fasanmade AA, Willerson JT (2003) Randomized, double-blind, placebo-controlled, pilot trial of infliximab, a chimeric monoclonal antibody to tumor necrosis factor-alpha, in patients with moderate-to-severe heart failure: results of the anti-TNF Therapy Against Congestive Heart Failure (ATTACH) trial. Circulation 107(25):3133–3140. 10.1161/01.Cir.0000077913.60364.D2 12796126

[178] Damås JK, Gullestad L, Aukrust P (2001) Cytokines as new treatment targets in chronic heart failure. Curr Control Trials Cardiovasc Med 2(6):271–277. 10.1186/cvm-2-6-271 PMC64832 11806813

[179] Nakamura M, Sadoshima J (2018) Mechanisms of physiological and pathological cardiac hypertrophy. Nat Rev Cardiol 15(7):387–407. 10.1038/s41569-018-0007-y 29674714

[180] Li L, Zhao Q, Kong W (2018) Extracellular matrix remodeling and cardiac fibrosis. Matrix Biol 68–69:490–506. 10.1016/j.matbio.2018.01.013 29371055

[181] Azevedo PS, Polegato BF, Minicucci MF, Paiva SA, Zornoff LA (2016) Cardiac Remodeling: Concepts, Clinical Impact, Pathophysiological Mechanisms and Pharmacologic Treatment. Arq Bras Cardiol 106(1):62–69. 10.5935/abc.20160005 PMC4728597 26647721

[182] Gibb AA, Hill BG (2018) Metabolic Coordination of Physiological and Pathological Cardiac Remodeling. Circ Res 123(1):107–128. 10.1161/circresaha.118.312017 PMC6023588 29929976

[183] Talman V, Ruskoaho H (2016) Cardiac fibrosis in myocardial infarction-from repair and remodeling to regeneration. Cell Tissue Res 365(3):563–581. 10.1007/s00441-016-2431-9 PMC5010608 27324127

[184] Packer M, Leptin-Aldosterone-Neprilysin A (2018) Identification of Its Distinctive Role in the Pathogenesis of the Three Phenotypes of Heart Failure in People With Obesity. Circulation 137(15):1614–1631. 10.1161/circulationaha.117.032474 29632154

[185] Adela R, Banerjee SK (2015) GDF-15 as a Target and Biomarker for Diabetes and Cardiovascular Diseases: A Translational Prospective. J Diabetes Res 2015:490842. 10.1155/2015/490842 PMC4530250 26273671

[186] Wollert KC, Kempf T, Wallentin L (2017) Growth Differentiation Factor 15 as a Biomarker in Cardiovascular Disease. Clin Chem 63(1):140–151. 10.1373/clinchem.2016.255174 28062617

[187] Farhan S, Freynhofer MK, Brozovic I, Bruno V, Vogel B, Tentzeris I et al (2016) Determinants of growth differentiation factor 15 in patients with stable and acute coronary artery disease. A prospective observational study. Cardiovasc Diabetol 15:60. 10.1186/s12933-016-0375-8 PMC4825089 27056183

[188] Berezin AE (2016) Diabetes mellitus related biomarker: The predictive role of growth-differentiation factor-15. Diabetes Metab Syndr 10(1 Suppl 1):S154–S157. 10.1016/j.dsx.2015.09.016 26482961

[189] Savic-Radojevic A, Pljesa-Ercegovac M, Matic M, Simic D, Radovanovic S, Simic T (2017) Novel Biomarkers of Heart Failure. Adv Clin Chem 79:93–152. 10.1016/bs.acc.2016.09.002 28212715

[190] Tsarouchas A, Vassilikos VP, Mouselimis D, Papadopoulos CE, Tachmatzidis D, Vassilikou A et al (2025) Iron deficiency in heart failure: cellular mechanisms and therapeutic implications. J Cardiovasc Dev Disease 12(11):415 10.3390/jcdd12110415 PMC12653584 41295341

[191] Alnuwaysir RI, Hoes MF, van Veldhuisen DJ, van der Meer P, Grote Beverborg N (2021) Iron deficiency in heart failure: mechanisms and pathophysiology. J Clin Med 11(1):125 10.3390/jcm11010125 PMC8745653 35011874

[192] Jurkowski F, Sito M, Marczak Z, Wójcik Ł, Gawron N, Bojanowski HT et al (2026) Iron Deficiency in Heart Failure: Pathophysiology, Diagnosis, and Therapeutic Strategies–A Narrative Review. J Educ Health Sport 89:69956

[193] Cunha GJ, Rocha BM, Falcão LM (2018) Iron deficiency in chronic and acute heart failure: A contemporary review on intertwined conditions. Eur J Intern Med 52:1–7 10.1016/j.ejim.2018.04.013 29680173

